# CompositeView: A Network-Based Visualization Tool

**DOI:** 10.3390/bdcc6020066

**Published:** 2022-06-14

**Authors:** Stephen A. Allegri, Kevin McCoy, Cassie S. Mitchell

**Affiliations:** 1 Laboratory for Pathology Dynamics, Georgia Institute of Technology and Emory University, Atlanta, GA 30332, USA;; 2 Department of Biomedical Engineering, Georgia Institute of Technology and Emory University, Atlanta, GA 30332, USA; 3 Machine Learning Center at Georgia Tech, Georgia Institute of Technology, Atlanta, GA 30332, USA

**Keywords:** CompositeView, link prediction, HeteSim, SemNet, biomedical knowledge graph, network analysis, concept relatedness

## Abstract

Large networks are quintessential to bioinformatics, knowledge graphs, social network analysis, and graph-based learning. CompositeView is a Python-based open-source application that improves interactive complex network visualization and extraction of actionable insight. CompositeView utilizes specifically formatted input data to calculate composite scores and display them using the Cytoscape component of Dash. Composite scores are defined representations of smaller sets of conceptually similar data that, when combined, generate a single score to reduce information overload. Visualized interactive results are user-refined via filtering elements such as node value and edge weight sliders and graph manipulation options (e.g., node color and layout spread). The primary difference between CompositeView and other network visualization tools is its ability to auto-calculate and auto-update composite scores as the user interactively filters or aggregates data. CompositeView was developed to visualize network relevance rankings, but it performs well with non-network data. Three disparate CompositeView use cases are shown: relevance rankings from SemNet 2.0, an open-source knowledge graph relationship ranking software for biomedical literature-based discovery; Human Development Index (HDI) data; and the Framingham cardiovascular study. CompositeView was stress tested to construct reference benchmarks that define breadth and size of data effectively visualized. Finally, CompositeView is compared to Excel, Tableau, Cytoscape, neo4j, NodeXL, and Gephi.

## 1. Introduction

The definition of “data visualization” can be as broad as the field itself. Per Tableau, one of many data visualization companies specializing in interactive visualization software, data visualization is “the graphical representation of information and data”, an admittedly broad, yet fitting, definition [1]. Friendly defines data visualization as “information which has been abstracted in some schematic form, including attributes or variables for the units of information” [2]. According to Ware, data visualization, as we know it today, is defined as “a graphical representation of data or concepts … an external artifact supporting decision making” [3]. For the purposes of this work, which develops the software tool CompositeView, data visualization is defined as the process of taking complex data and representing it in an interactive, composite manner. As suggested by the tool name, a composite view effectively mitigates information overload, which otherwise clouds actionable insight.

A large number of data visualization tools exist. Some are more general-purpose, such as Microsoft Excel and Tableau [4], and others are more specific, such as the network visualization program, Gephi [5]. Many programs are available to simply visualize data where a user predefined the attributes. However, some data sets are so large and complex that simply visualizing data under predefined attributes results in information overload; that is, there is simply too much data to meaningfully acquire actionable insight. For example, imagine a network with so many nodes and edges that it simply becomes an entanglement of chaotic lines on the screen. Manual filtering or post-analysis can assist, but it is a slow, tedious process that requires multiple subjective decisions by the domain user.

Here we develop CompositeView, an open-source Python-based data visualization tool, to assist domain specialists in deriving actionable insights from large, complex data sets that can be visualized in network form. CompositeView was originally inspired by link prediction and relevance scoring methods [6], but was generalized for a greater breadth of data. The key gap filled by CompositeView is the ability to automatically calculate and update composite scores as the user simultaneously interacts with the data. Composite scores are defined representations of smaller sets of conceptually similar data that, when combined, generate a single score. Interactive composite scoring and corresponding visualization greatly decreases information overload in complex network or non-network data, enabling effective and efficient actionable insight [7]. Interactive composite scoring also provides a pivotal quantitative metric to further optimize user filtering and visualization. By contrast, built-in interactive composite scoring is not currently available in other generalist visualization tools or even specialist network visualization tools.

The remainder of the Introduction section provides the necessary background, motivation, and design criteria for CompositeView. The Methods section details how CompositeView was constructed and how it is used, including a stress testing analysis for CompositeView’s data handling, processing, and efficiency. The Results section illustrates three distinct data set examples visualized with CompositeView: results from a SemNet [8,9] biomedical knowledge graph analysis of the two target nodes AD and hypothyroidism, Human Development Index (HDI) data [10], and cardiovascular disease (CVD) data [11]. Two of these three examples are completely removed from the study of networks, and the example involving HDI data is not strictly related to biomedical sciences. The choice of these data is intentional, as they are both simple to understand and useful for showcasing the potential power of CompositeView as a flexible and accessible visualization tool for disparate domains. The Results section also illustrates baseline stress testing of CompositeView’s data handling and processing speeds. The Discussion compares CompositeView to other visualization tools and elaborates on limitations and future directions. Six visualization tools ranging from least to most similar to CompositeView are compared and contrasted: Microsoft Excel, Tableau, Cytoscape, neo4j, NodeXL and Gephi.

### 1.1. Background

The field of computational biology has grown tremendously in the last 50 years, due in many parts to the growth of both computational power and vast biological data sets [12,13]. The culmination of these advancements peaks at the intersection of biology and network analysis, where biological systems are investigated through the use of networks and graph theory [14]. Generally, the networks studied in computational biology model discrete biomedical entities as nodes/vertices/points and the relationship between entities as edges/links/lines [15]. These networks can be broadly categorized as either undirected or directed graphs, where directionality is determined by whether the ordering of a connected pair of nodes is significant. Networks can also have weight values associated with edges, where the weight will often indicate the relevance of the pairwise node connection, and degree values associated with nodes, where degree (or in-degree and out-degree, with directed graphs) corresponds to the number of edges a node has.

Given the high degree of integration between computational biology and network science, the abstract nature of biological networks has been condensed into more concrete network types that have specialized uses. A few of the many types of biological networks include protein–protein interaction networks, genetic interaction networks, metabolic networks, drug-target networks, and literature co-occurrence networks, among others [16]. The first layer of insight from these networks can be obtained through both their properties as graphs and their overall structural patterns. The observation of these properties and patterns, such as graph density, graph isomorphism, clustering coefficients, node centrality, network motifs, and others, can, in many ways, provide valuable insight on the internal organization of a biological network and elucidate the constraints responsible for the functional protein, as well as metabolic, regulatory, and genetic systems observed in nature [17,18]. More nuanced approaches used for gaining insight from networks include the utilization of network-based modeling, ranging from network-structure-oriented methods, such as network centrality, network propagation, and structural similarity-based link prediction, to machine learning (classification, clustering, etc.) and deep learning (neural network) algorithms.

Link prediction and similarity scoring, methods which are used for discovering potential relationships between objects within a network exist at the very cutting edge of biological network science and have a variety of real-world applications. Link prediction is the catch-all term for analytical tasks that can reveal unknown relationships between entities in a graph with some level of quantifiable confidence. The existence of proposed edges can be determined using a variety of methods, such as topological similarity measures (using the way nodes and edges, along with inherent properties, are specifically arranged within a network to gain insight) such as the Jaccard Index [19], the Sorensen Index [20], the CAR-based Common Neighbor Index [21]; cosine similarity, probabilistic, and maximum likelihood methods, such as those proposed by Wang et al. [22], Yu et al. [23], and Guimerà et al. [24]; and embedding-based methods, such as DeepWalk [25], Node2vec [26], and HARP [27,28]. One application of link prediction, drug discovery, is the practice of using networks to discover potential connections between drugs and other drugs, proteins, diseases, genes, or alternative concepts of interest [29]. For example, link prediction was recently used to discover and suggest repurposed drugs for the novel COVID-19 virus using data from older coronavirus pandemics and a small subset of COVID-19 data to identify similarities [6]. Another practical example of link prediction and relevance measures in action comes from the analysis of literature co-occurrence networks and knowledge graphs. These networks are built from relationships between entities extracted from text corpora, large bodies of structured text such as PubMed, PubMed Central, or Wikipedia [17]. Literature-based discovery (LBD), a field that aims to extract insight from existing literature-based sources and generate new connections between existing knowledge, is a prime example of an area of study that can use literature co-occurrence networks, knowledge graphs, link prediction, and relevance measures to great effect.

### 1.2. Motivation

SemNet is a software tool developed to conduct LBD on an expansive heterogeneous information network (knowledge graph) constructed from the Semantic Medline Database (SemMedDB) [8,9]. This database is, essentially, a table containing text-mined subject-predicate-object triples, extracted from article abstracts within PubMed, that convey some sort of relationship between biomedical concepts [17]. Given the expansive nature of PubMed, the knowledge graph used by SemNet is quite large, at approximately 20,000,000 edges and 300,000 nodes (where nodes represent concepts and edges represent relationships between concepts). SemNet enables LBD by employing the power of the HeteSim similarity metric, a metapath-based relevance measure used to determine the similarity of two objects within a heterogeneous information network [30]. The practical implication of this is, given two concepts (referred to as nodes) within the SemNet knowledge graph, the relatedness of these nodes can be computed and compared. In practice, SemNet has been used for hypothesis generation by first identifying a node of interest within the knowledge graph (target node), then determining all connected nodes (source nodes) of some specified distance away from the target within the graph, and finally ranking these source nodes in respect to the target node by their aggregate HeteSim similarity score. As a brief aside, because HeteSim is calculated on a per-metapath basis, and source and target nodes are often connected by multiple metapaths, the aggregation of HeteSim scores is necessary for a combined relevance value derived from multiple metapaths. This aggregation is typically the arithmetic mean of all HeteSim values; however, other aggregation methods (specifically randomized aggregation, in SemNet 2.0) have been used successfully [9].

As practical studies using SemNet become more complex, a new problem involving complexity creep and information overload arises: the results of network analysis can become overly difficult to interpret for practical use. This is a phenomenon that can be observed when combining multiple uses of SemNet into a single, comprehensive analysis of multiple targets of interest. Take the combined study of Alzheimer’s disease (AD) and hypothyroidism, for example. Each run of SemNet will identify a set of source nodes of a specified distance away from a target node within the knowledge graph and calculate the aggregate HeteSim score for each source–target pair. Each node in the set of connecting source nodes now has a relevance measure associated with a specific target, which allows the sources to be ranked. To instead study the source nodes that are connected to both targets at once, an intersection of the two sets of previously discovered source nodes is taken, and the new set of nodes is ranked against the two targets, respectively. This generates two sets of results that, when studied directly, could take valuable time to analyze and gain actionable insight. CompositeView, the main tool described in this work, is a data visualization software that aims to improve the interpretability of such data by translating algorithmic discovery into actionable insight using the power of network visualization techniques and interactive data visualization.

To show how CompositeView realizes this definition of data visualization, the proposed example described above involving the SemNet analysis of AD and hypothyroidism will be continued. In this example, the SemNet results are visualized as a new graph where source and target nodes are related by the newly proposed connections (modeled as edges). The aggregate HeteSim score associated with each source and target connection is assigned as an edge weight in the graph. The two aggregate HeteSim scores associated with each source node in the set of shared source nodes (one score for each source–target relationship, of which there are two per source node) are combined into a single value. This value is the composite score, which is subsequently assigned to the source node in the visualization. How this composite score is calculated depends on the context of the results being visualized. In terms of SemNet, the composite score is the arithmetic mean of all outgoing edges connected to each source node. This composite score condenses information into a joint metric that, though less informative overall, can convey insight from the data in a way that justifies the drawbacks of minimal information loss. In the context of the two targets in the example above, the composite score associated with each source node conveys its relatedness to both AD and hypothyroidism simultaneously. Ultimately, the graph, edge weights, and composite score come together to form the backbone of CompositeView and define its core approach to visualizing data. This foundation is subsequently expanded upon using network layout algorithms, interactive components, and a web application framework to generate a comprehensive data visualization tool.

Beyond SemNet, other network analysis methodologies can encounter situations where the communication of data needs improvement. For example, take the study conducted by Timilsina et al. where a heat diffusion algorithm is applied to a two-layer network created using genetic interaction databases (STRING and BioGRID) to discover potential associations between tumor samples and genes [31]. An example scenario where CompositeView could be helpful is when multiple genes are potentially predicted for multiple tumors. If researchers want to take their analysis a step further and understand how each gene interacts with multiple tumors using a single value, a composite score methodology could be formulated and applied to each source node. The integration of CompositeView into the existing network analysis pipeline could prove useful for communicating the large amount of data produced whether for hypothesis generation or validation.

Another instance where CompositeView could improve network analysis pipelines comes in the form of DICN, a similarity-based link prediction method described in Zareie et al. [32]. Though not strictly a tool that generates a ranked list of nodes (such as SemNet), other pipelines that implement DICN could conceivably use it for this purpose. For practical applications of DICN where multiple sets of nodes are compared to multiple individual nodes, especially when studying social networks, ecological networks, airport traffic networks, or co-authorship networks (all experimental networks mentioned in Zareie et al.), among others, the integration of CompositeView could reduce the time required to interpret results as well as introduce an interactive element that expands the potential for insight from the results themselves.

As a final practical use case, take the Optimal Vascular Care measure [33]. This is a composite score of four patient goals that, when reached together, represent a gold standard for managing vascular disease. Though not strictly a network-based method, the practical use of the Optimal Vascular Care measure could take advantage of CompositeView by modeling patients as source nodes, the four goals as target nodes, the adjusted measurements as edge weights, and management goal status as node groupings.

### 1.3. Design Criteria

During the design process of CompositeView, key features were noted and will be listed in order from most to least important in the design hierarchy. These key features are a result of experimenting with multiple ways of visualizing composite score data (using different types of charts, graphs, etc.) as well as general SemNet user feedback. CompositeView was originally designed to visualize SemNet results, so users of SemNet would suggest features that would make the interpretation of results more intuitive. This feedback was then expanded upon after generalizing CompositeView, and additional feedback was used in feature selection when discussing how to visualize more general composite score data.

First, CompositeView provides clean, easily readable data visualization; with it being a visualization tool, ensuring the data is communicated effectively is the first priority. CompositeView originates from the study of networks, so adopting common network visualization techniques, such as node and edge representation, node and edge sizing, and aesthetic network layouts is a natural translation for communicating the data (as opposed to more standard plotting). Network layout is a key problem in network visualization, and because classic network layout algorithms perform relatively poorly when visualizing disparate clusters of nodes individually connected to multiple targets, a new method has been formulated for CompositeView’s specific data input.

Next, CompositeView is designed to accommodate multiple sets of data (originally in the form of SemNet results), so a composite scoring functionality is key for communicating the overall relatedness of data points. Composite scores, in CompositeView’s current state, are calculated using the arithmetic mean of all outgoing edge weights associated with each respective source node in the network. The use of arithmetic mean (as well as the source and target node terminology) is an artifact of CompositeView’s SemNet origins; however, the visualization of HDI and CVD data described in later sections specifies how the combination method can be altered based on the data and ranking criteria.

Third, CompositeView expands upon the provided data by being interactive, specifically through the use of a filtering and graph manipulation functionality. Filtering entails node, edge, and type (nodes can be assigned types or groups) selection by employing range sliders and dropdown components. Range sliders allow value bounds to be set that enable or disable the inclusion of nodes and edges based on their assigned value (composite score and edge weights, respectively), and dropdowns allow specific named nodes and types to be selected for unique visualization. Changing the inclusion of edge information specifically will change the composite score associated with each source node. Graph manipulation encompasses all interactivity that facilitates customized visualizations through color changes, layout adjustments, simulation iterations (which affect network layout), and node and edge sizing.

The final design criterion is that CompositeView be both accessible and flexible for a variety of unique situations. This final feature is less tangible, but a necessity, nonetheless. During software development, specific tools were chosen because they are inherently well integrated and simple to use. CompositeView has an internally modular design, which gives it the ability to exist in a relatively niche data visualization field while still maintaining flexibility. Accessibility and flexibility are what allow CompositeView to integrate itself into a variety of data pipelines. These two core tenets will be the foundation for future development and scope expansion.

## 2. Methods

At a high level, CompositeView’s objective is to take formatted input data and communicate it using both graph (network) visualization methods and interactive components. These data follow a specific format, described in detail in Section 2.1.3, and generally consist of items/data points/“things” ranked or related to concepts/metrics/“things”. These data are modeled as nodes, specifically source nodes (the items being compared) and target nodes (the items being compared against). These nodes are connected by edges, which are weighted based on the value shared between a source and target. This value can be a ranking, probability, relevance, likelihood, or any quantifiable value that relates two nodes. The input data also have types or categories assigned to each node, both source and target, that allow these nodes to fall into discrete buckets. Finally, the input data imply directionality; edges point away from source nodes and towards target nodes.

The output presented by CompositeView consists of a visualized network where source nodes are connected to their corresponding target nodes by edges. Both the source and the target nodes are colored by their respective types (“buckets”), and the size of source nodes is determined by each node’s composite score. This composite score (combined value) is a combination of all outgoing edge weights assigned to each source node; the combination method itself, whether an arithmetic mean, geometric mean, joint probability, etc., can be adjusted by the user within the source code. The visualization presented by CompositeView can be filtered based on edge value, combined value, node type, and edge name, and the visualization itself can be adjusted through node and edge sizing, color mapping, node spacing, and target spacing. The framework application for CompositeView can be hosted either locally or externally, and custom data can be integrated either through an upload button or through the source code itself.

### 2.1. Developing CompositeView

#### 2.1.1. Choosing the Appropriate Tools

CompositeView aims to be as accessible and flexible as possible. Accessibility, as the first of these two core pillars, is achieved by reducing the amount of technical prerequisites necessary to work the tool. If a user knows the limits (Section 4.2) and scope (Section 4.3) of CompositeView, and the data fit the correct input format, then using the tool itself should not be a bottleneck. The second of the two pillars is flexibility, an advantage that CompositeView potentially has over other data visualization tools. Flexibility, in this case, is defined as the ability to quickly and easily evolve CompositeView, under reasonable constraints, to fit the context of the data being visualized. If a potential user of CompositeView has data that fits the general data input format, but the tool itself does not quite provide the desired outcome, that user, with as little prerequisite knowledge as possible, should be able to make adjustments that suit their particular use case. At the intersection of accessibility and flexibility, the tools used to build CompositeView are chosen. Creating a foundation that promotes these two core tenets is essential for CompositeView to succeed, and choosing the appropriate tools that can build this foundation is equally crucial.

To start, CompositeView exists within the definitions of data visualization that include computational support—physical drawings can be useful, but their intractability is limited. To achieve the flexibility desired in CompositeView, it must be built using a programming language; this choice produces a trade-off that decreases accessibility in favor of flexibility. It is possible, perhaps even common, to utilize multiple languages when developing a tool similar to CompositeView, but in order to maintain as much accessibility as possible, another trade-off in favor of accessibility (at the loss of flexibility) has been made. A single programming language, famous for its ubiquity and ease of use, has been chosen as the main driver of CompositeView: Python (specifically Python version 3.9.7) [34]. According to the 2021 Stack Overflow Developer Survey, contributed to by over 80,000 developers, Python is currently the third most popular programming language on the platform (and the most popular language outside of dedicated web development) [35]. Python is a general-purpose language with a focus on code readability and clear syntax that exists at the forefront of scientific computing, particularly in the fields of machine learning, artificial intelligence, biology, and many others [36]. Python also contains a plethora of useful statistical, analytical, and mathematical libraries that provide all of the functionality that CompositeView requires (and more, for others to explore).

With Python as the foundation for CompositeView, certain key building blocks are chosen to fulfill integrated roles. NetworkX, a Python package for the creation, manipulation, and study of complex networks, is the first of these building blocks and the core of CompositeView; it is used to model the data itself [37]. To reiterate, CompositeView presents data using concepts from graph theory and network analysis. Source nodes model data points, target nodes model entities of interest or comparison metrics, and edges model the quantified relationships between the two. NetworkX allows for the efficient creation of graph objects that can be used to store data, simulate layouts, and run algorithms (such as a modified version of depth-first search) that allow for key graph features to be efficiently computed. The Visualizer utilizes NetworkX to both store the graph and help simulate the layout, both of which are integral to its overall function.

To give CompositeView a scaffold to both display and interact with the graph itself, Dash, a low-code framework for rapidly building and deploying web applications, is the second of the three major building blocks [38]. Dash is built upon Flask, Plotly.js, and React.js and enables the development of full stack web applications using only Python (along with some HTML, CSS, and JavaScript when desired). Dash has been chosen as the framework for CompositeView due to both its simplicity and its ability to abstract complicated technologies and protocols away in favor of a streamlined approach to development. The choice to use Dash is another trade-off between flexibility and accessibility, this time in favor of accessibility, that helps maintain the homogeneous and Python-driven theme of CompositeView. Knowing Python, and having the ability to interpret Dash documentation, are the only two prerequisites (up to this point) that are required to make custom changes to CompositeView. Dash provides HTML components, CSS styling, and interactivity through callbacks to CompositeView, along with sliders, drop-down menus, buttons, tables, and other features that provide a framework for data manipulation and viewing. Interactivity is what ultimately elevates CompositeView beyond simple graphical representations of data; it allows for novel insights and new perspectives that would otherwise be very difficult to produce. Dash is what provides the tools that make this interactivity possible.

The third and final of the three major building blocks employed by CompositeView is Cytoscape, specifically Dash Cytoscape [39]. Dash Cytoscape is a graph visualization component of Dash that specializes in creating customizable, high performance, web-based networks rendered using Cytoscape.js. Dash Cytoscape is deeply integrated with the Dash framework, enabling many of the key features that make Dash especially useful work seamlessly with the displayed graph. Dash Cytoscape is the tool that unifies Dash and the NetworkX graph object; with some simple manipulation, the graph object can be converted into a Python list of elements that Dash Cytoscape renders and displays in the Dash application. These three tools have been carefully selected due to, in many parts, their ability to align with the two pillars of accessibility and flexibility that have been key in the design philosophy of CompositeView. Other key packages, such as pandas and NumPy, are integral to CompositeView and used throughout the code [40]. Pandas, arguably the most important of the two for CompositeView, is a fast, powerful, easy-to-use open-source data analysis and manipulation tool built on top of Python [41,42]. Much of CompositeView’s data is stored within pandas DataFrames, and the speed and efficiency of these DataFrames, in part a product of using NumPy under the hood, are invaluable in the creation of CompositeView.

#### 2.1.2. The Graph–Application Split

**Figure 1.**
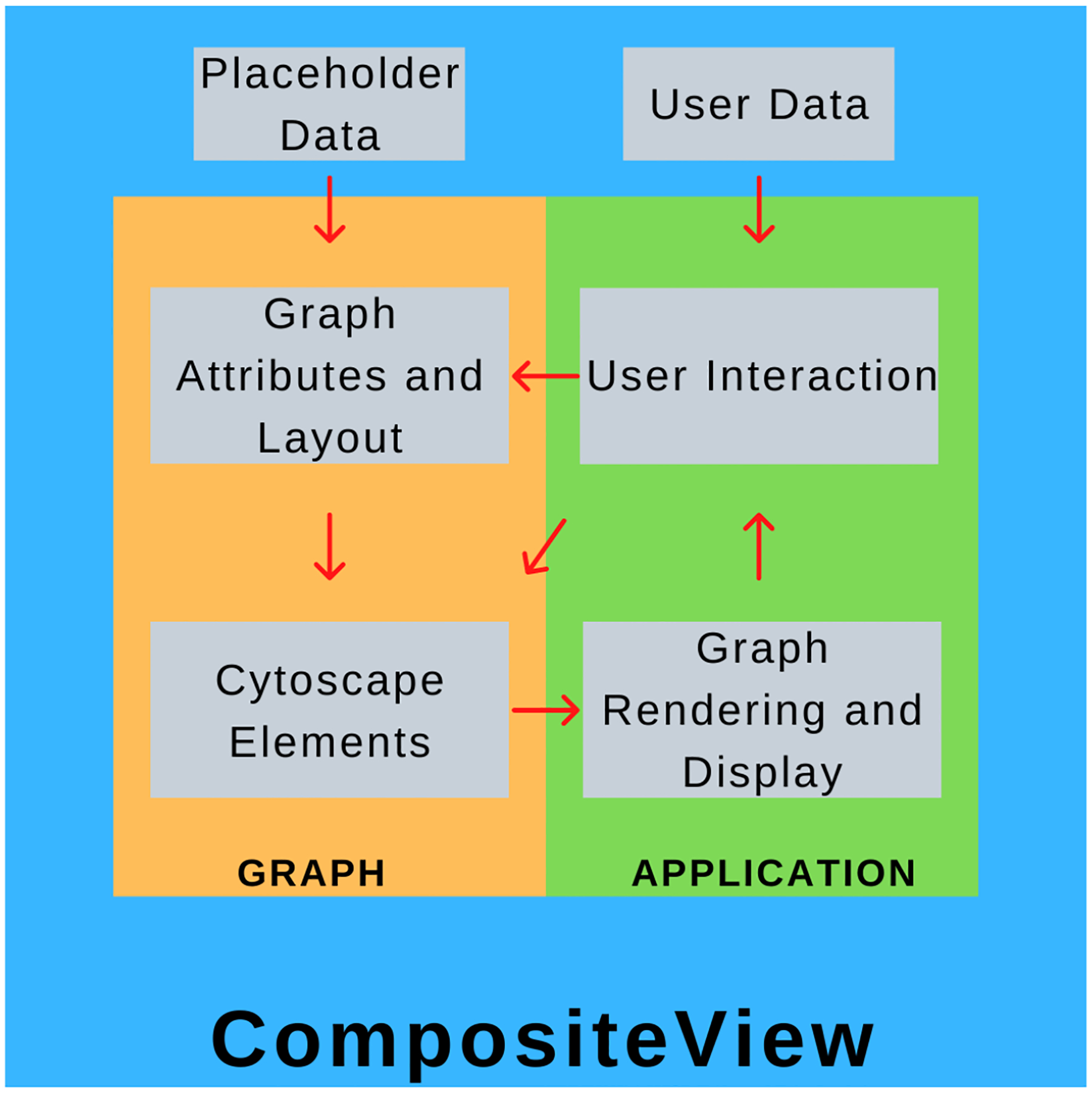
A high-level overview of CompositeView’s working cycle. CompositeView has placeholder data, which initialize the graph, and user data, which initialize the user interaction and application of CompositeView. The cycle begins with a user uploading data and interacting with the application to update the graph attributes and layout. Next, the Cytoscape elements are updated to run the graph. Finally, the graph rendering and display are visually updated to the user in the CompositeView application. The working cycle continues as the user makes updates to the data or changes or applies CompositeView application features such as graph layout selection or filtering modes.

CompositeView is conceptually split into two parts: the “graph” component and the “application” component. Both of these parts are isolated within their own respective Python scripts and communicate through a class instance, referred to as the graph object. The graph component centers around a class that initializes and stores the graph attributes and metadata, simulates the layout, and generates the element list that is eventually fed to Dash Cytoscape. An instance of this class is created within the application script, where the page layout is defined, user interface elements are constructed, and Dash callbacks are built. When the application script is run, a web server is started (Dash is built upon Flask, a micro web framework written in Python) to display the graph, interactive elements, and data outputs (spaces displaying node data and tabular data) [43]. These interactive elements are Dash HTML components which interact with callbacks which, in turn, interact with the graph object itself, updating the graph object if applicable, which updates the displayed graph and interactive element states. This cycle is repeated until the application is terminated. Ultimately, the application (and elements contained within it) is the tool that allows the user to directly modify the graph object, and the graph object is what the application uses to display information about the data. A high-level overview of this process can be seen in Figure 1.

#### 2.1.3. Data Formatting and Input Structure

**Figure 2.**
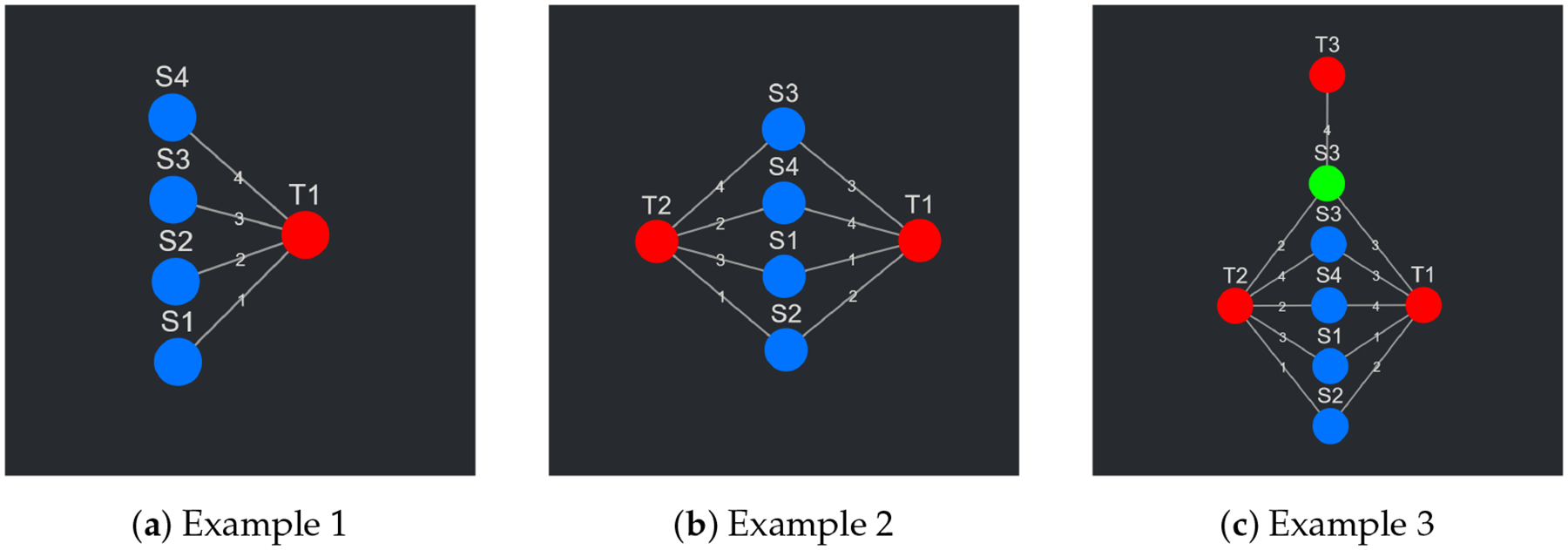
The three input data examples explained in Section 2.1.3, evolving from least to most complex.

To understand the data input format, the data themselves, the data that Composite-View is built to visualize, must first be described. To begin with an abstract example, in its most basic form, these data consist of a set of items. These items are ranked in respect to some arbitrary concept, metric, or “thing” (biological entity or otherwise) based on a quantitative value (probability, likelihood, etc.). The items, or data points, in the set can be modeled as nodes (termed source nodes), the arbitrary concept, metric, or “thing” can also be modeled as a node (termed a target node), and the quantitative value shared between a source and target node can be modeled as an edge, where the value itself is the edge weight. To better conceptualize the current state of this example, see Figure 2a. The data abstraction just described is, in its most basic form, the data that CompositeView can visualize. To solidify this abstraction further, a snippet of a sample table is shown below in Table 1. Each row corresponds to a unique source–target relationship, and each column represents the source node name, the target node name, or the edge value. This is not the final layout, but a first step in the building of it.

As a second example, take the data described above where, instead of a single target, the same set of source nodes is related to two targets, each with different edge values (see Figure 2b). Each source node now has two edges, one for each target relationship. This is where the concept of a composite score makes its first appearance. Each source node, with its two edges, can join these values into a single combined value in a variety of ways (arithmetic mean, geometric mean, weighted sum, joint probability, etc.). This composite score, depending on the context, might hold some significance—this will be touched upon later. This new abstraction is again further solidified in Table 2, where 8 rows correspond to 4 unique source nodes, each with two relationships with two respective targets. This is closer to the final data input format, but not quite there.

As the final and third example, take the set of all nodes (both source and target) as described above and expand it to include another two nodes: a source node and a target node. The target node is completely unique; however, the new source node shares the name with an already existing source node. This will prompt each source node to have a new value associated with it: a unique identifier. This unique identifier allows source nodes with the same names (but potentially different targets) to exist as separate entities within the network. To continue this example, the newly added source node shares two targets with its shared name partner. It also shares an edge with the newly added target node. See Figure 2c to see the visual representation of this abstraction. To potentially contextualize the new source node, all the source nodes are assigned a discrete category. The original group of source nodes is categorized under “G1” while the single new source node exists in “G2”. Realistically, these groups could represent an infinite number of things, but in this situation the grouping exists simply to delineate source nodes that connect to different sets of target nodes. Table 3 shows how this abstraction is formatted into a table. This table is, in fact, the final data input format for CompositeView.

In Table 3, each row corresponds to a source–target relationship within the graph. Each source and target node are assigned a unique identifier, name, and type (group), and both share a single edge value. All three examples described above, from the simplest to the most complex, can be represented in this table. To show this, take the first and simplest example described above involving a set of source nodes ranked in respect to a single target node. Table 4 below shows how the CompositeView input data format could accommodate for this situation. In more precise terms, the columns and data types for the input data format (structured as a table) are as follows: source_id [text], source_name [text], source_type [text], target_id [text], target_name [text], target_type [text], edge_value [numerical]. The data are accessed by CompositeView either directly as a pre-formatted DataFrame used as a graph class argument or as a formatted Comma Separated Value (CSV) file that is uploaded (and subsequently converted into a DataFrame) using a button component within the application itself. CSV files do not hold data type information; however, when the file is read into CompositeView’s edges DataFrame, the columns are cast as the appropriate types. A variety of formatted data table examples used as inputs for CompositeView are provided in the associated GitHub repository.

#### 2.1.4. Graph Layout Methodology

**Figure 3.**
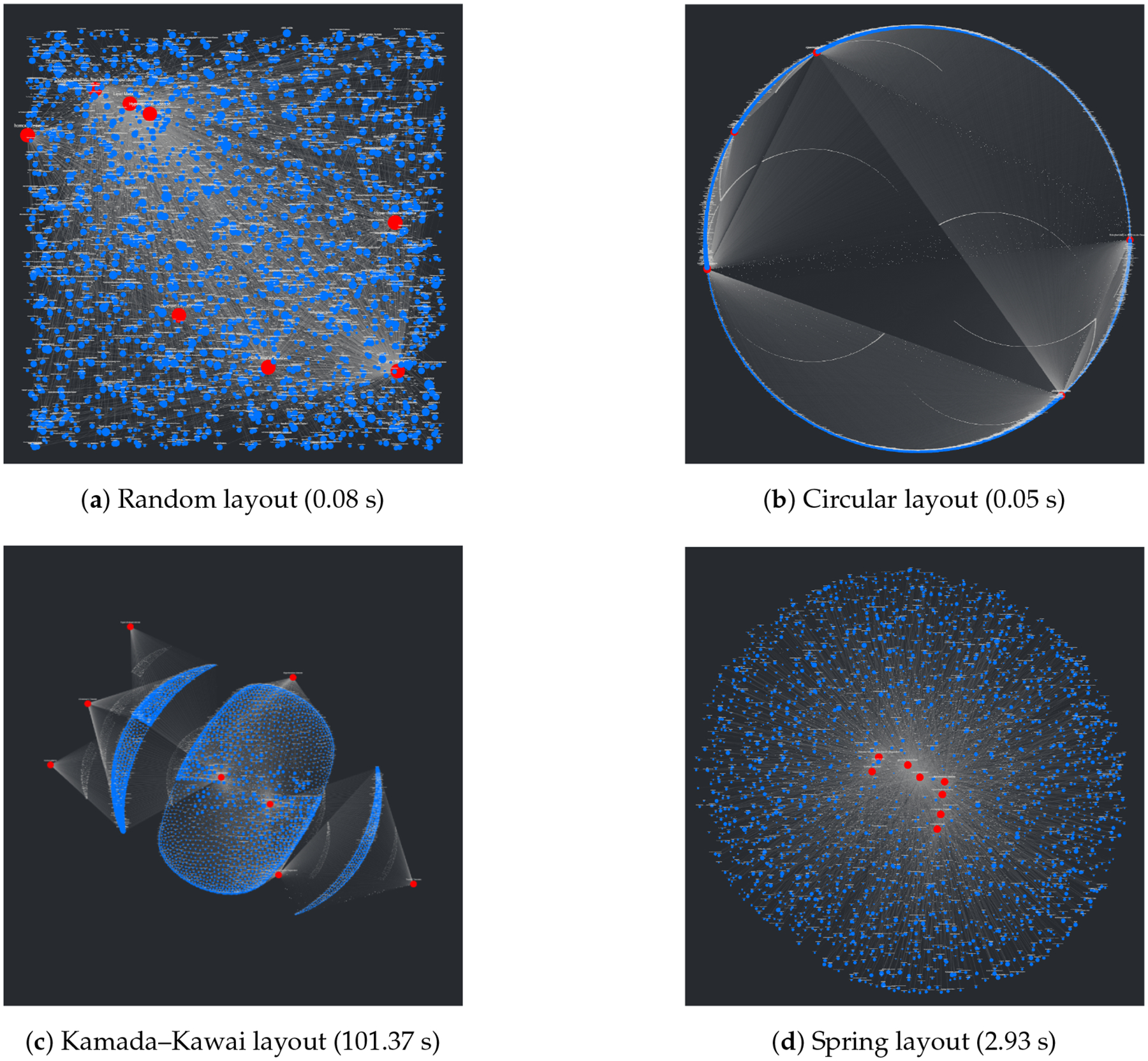
A sample of tested graph layouts along with their CompositeView runtimes, all based on the same SemNet results data set (approximately 2472 source nodes).

An interesting problem regarding the visualization of networks involves the process of placing nodes and edges to maintain an aesthetic and clear layout. At a surface level, this seems trivial and underdefined, but given that the main purpose of CompositeView, and visualization tools in general, is to communicate data succinctly and clearly, poor layouts can prove to be extremely detrimental to this goal. Using the NetworkX package, many graph (mathematically defined network) layout algorithms are easily available. A list of layouts tested with CompositeView include the random layout, circular layout, Kamada–Kawai layout (based on the Kamada–Kawai path-length cost function), and spring layout (based on the Fruchterman–Reingold [FR] force-directed algorithm), along with a handful of others [44,45]. Figure 3 shows a visual comparison for each of these layouts, along with their respective runtimes, created using a typical combination of SemNet results. In this figure, target nodes are red and source nodes are blue, regardless of true categorization. The runtime is measured across the entire method responsible for creating the layout, which includes data preprocessing and other operations associated with layout generation. Ultimately, the Kamada–Kawai layout resulted in the most clear and discernible node separation of all the preset algorithms presented by NetworkX; however, a relatively long runtime (given this typical data set) makes it impractical, even with tuning algorithm parameters. The second clearest layout, the spring layout, is a step below the Kamada–Kawai layout visually (even when tuning parameters, such as node repulsive force and edge weight); however, it runs much faster given the SemNet data sets used for testing. The layout itself is not especially appealing, but with some adjustments in how node positions are initialized and fixed, the spring layout becomes the ideal algorithm for generating graph layouts for CompositeView.

**Figure 4.**
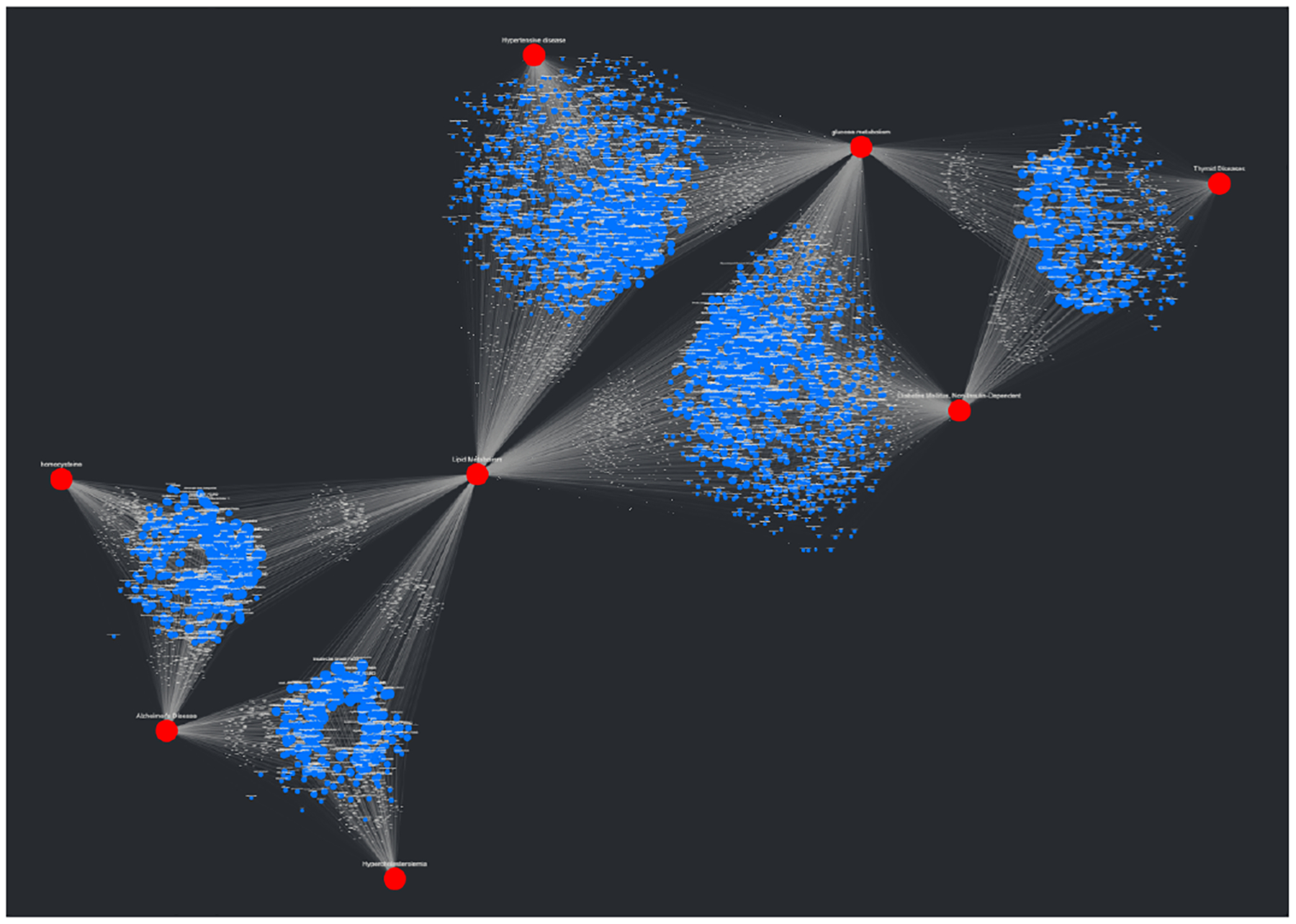
The adjusted spring graph layout using the same SemNet test data from Figure 3 (runtime: 5.78 s).

A major problem with the presented layouts is that target nodes often become “stuck” within large clusters of source nodes, losing the potential to quickly convey useful information about which source nodes are clustered and connected to these targets. This problem can be observed in essentially every layout shown in Figure 3; target nodes can be easily found (with the help of sizing and coloring), but knowing which source nodes connect to them is nearly impossible. This problem is simply an artifact of how force-directed graph drawing algorithms, specifically the Kamada–Kawai and Fruchterman–Reingold (spring layout) algorithms, function. Both of these algorithms define an objective function that, in general terms, utilizes attractive and repellent forces (which are defined differently between algorithms) to generate an overall energy value for the graph, which is subsequently minimized. With “larger” graphs, like those presented in Figure 3, the objective function is more often than not minimized locally, producing a layout with an overall energy value higher than the global minimum [46]. This results in layouts that can be, for lack of a better term, unpredictable. To combat this unpredictability, while also isolating both target nodes and source node clusters, an adjustment to the spring layout was made, roughly based on a multi-scale technique. This “adjusted spring layout” methodology aims to generate a rough abstraction of the graph, then build upon it in a layered fashion with increasing detail. A preview of this methodology in action, using the same SemNet data as Figure 3, can be seen in Figure 4.

**Figure 5.**
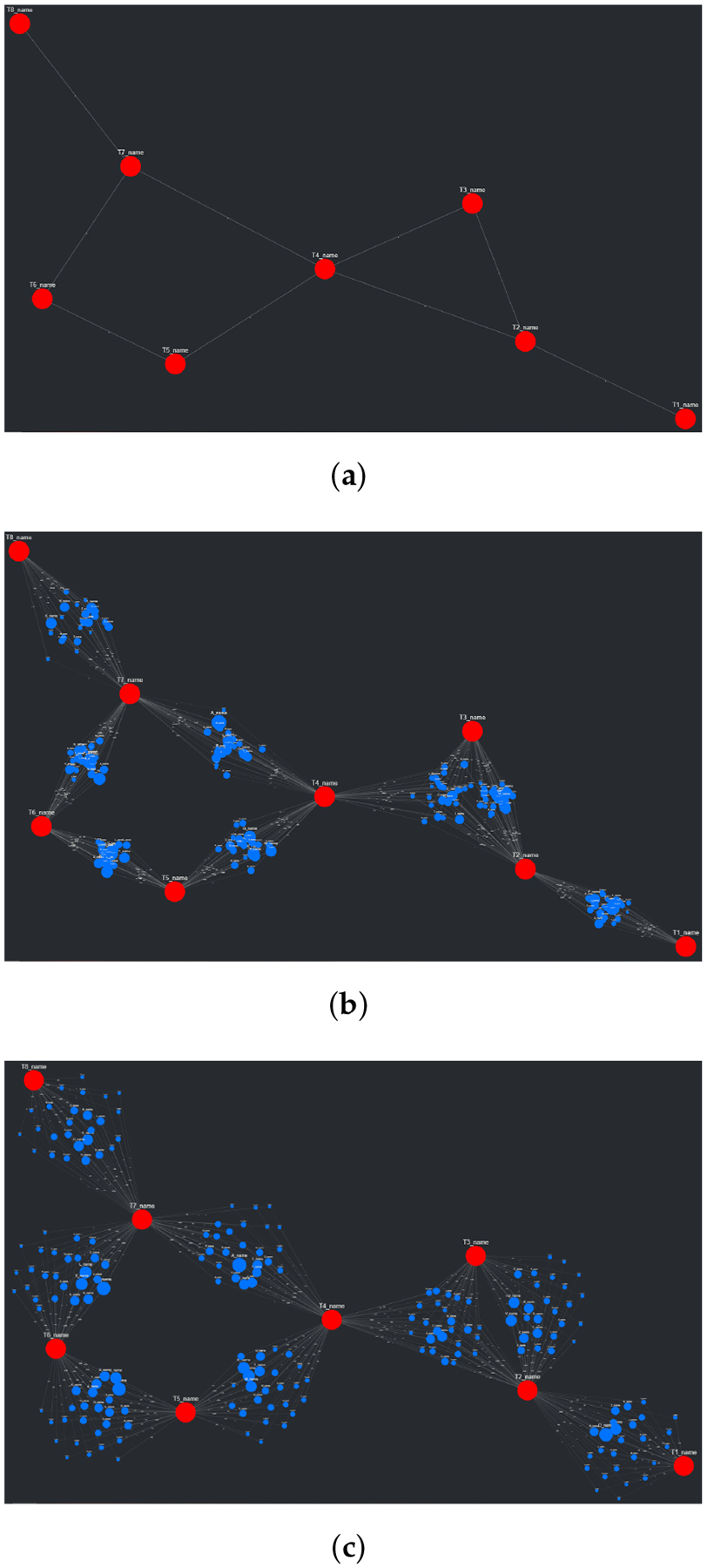
The adjusted spring layout method, broken down into three logical steps. The data shown are placeholder data used in CompositeView. (**a**) Initial target nodes are simulated and positions are fixed. (**b**) Artificial edges are removed and source nodes are filled in around the shared target node centroids. (**c**) Source nodes are simulated with edge weights.

The first of three parts in the adjusted spring layout process involves first isolating the target nodes, then simulating them using the FR algorithm. In this first step, the targets are connected using artificial edges. These edges are based on whether intermediate source nodes connect a set of linked targets. For example, if a set of target nodes all connect to a shared set of source nodes (a cluster), they themselves will be connected with shared artificial edges. This example scenario is quite common with SemNet results, given that often they involve a single set of source nodes being ranked in respect to multiple targets. The weights associated with the artificial edges (weight values in the FR algorithm increase or decrease the “pull” of an edge on a node) are inversely proportional to the number of source nodes connected to a shared set of targets. If the previous example set of target nodes are connected to the same 80 source nodes, the artificial edge weights connecting them to each other would be less than if the same set of target nodes were connected to the same 40 source nodes instead. This might seem counter-intuitive, having weight values increase with a decrease in connectivity, but this choice results in more “room” between targets for the eventual source nodes to be placed and subsequently simulated. Once the target nodes are simulated (in this case, using 50 iterations of the FR algorithm), their position is fixed. To better understand this first portion of the adjusted spring layout method, see Figure 5a.

The second part of this three-step method involves re-populating the visualization with the briefly removed source nodes. Now that all target nodes have their position fixed, the artificial edges are removed, and the source nodes are placed once again on the graph. Each group of source nodes that share common targets are placed around the centroid of these targets following a Gaussian distribution (if all the source nodes were placed on the centroid itself, they would have no repelling force). See Figure 5b to better understand the initial source node placement. With the source nodes “filled in”, the third and final of the three steps is enacted, starting with running the FR algorithm once again, though with (recommended) fewer iterations (see Figure 5c). The algorithm time scales with the number of nodes in the graph as discussed in the results presented in Section 3.1. Keeping the iteration count low will improve runtime at the cost of the potential interpretability of the graph. This is a trade-off that users can make themselves, using the Graph Manipulation elements provided in CompositeView.

#### 2.1.5. Composite Scores

A unique feature of CompositeView is its ability to calculate composite scores for every source node in the graph. Composite scores can be defined as representations of small sets of conceptually and statistically similar data that, when combined, generate a single score with the potential of reducing information overload [7]. This composite score, in CompositeView’s current state, consists of the arithmetic mean of all edge weights “leaving” each source node, an artifact CompositeView’s SemNet origins. However, the combination method used could be easily edited if the data set being visualized calls for it. Anything from simple weighted linear combinations to more complex joint probabilities and functions could be used to combine outgoing edge values on a per source node basis. Understanding the benefit of calculating composite scores is best shown with an example.

Take the data set for the Human Development Index (HDI) [10]. The HDI is a composite score that aims to quantify the “development” of a country, based on three specific indices: the life expectancy index, the education index, and the income index. Each of these measures aim to capture different aspects of what make a country developed, but, for CompositeView, the most important application of this data set is that it simply and clearly captures how composite scores (and the visualization of such scores) are useful. Each country analyzed for an HDI ranking can be modeled as a source node. These 189 countries are ranked in respect to three metrics which combine to create the HDI composite score. In CompositeView, each country, as a source node, shares three edges with the three index values, each modeled as a target node. The HDI value is calculated by taking the geometric mean of the three index values per country. An example of HDI data displayed using CompositeView can be seen in Section 3.2.2. The Python class that creates the graph object (the object that is directly manipulated by the application and generates the element list for Dash Cytoscape to read) contains a method dedicated to combining values and generating the composite score for every source node. The main pandas DataFrame that contains all of the graph edge information is filtered to create a unique sub DataFrame for each respective source node. This sub DataFrame is fed to the value combination method which, in turn, can use any information in this sub DataFrame to calculate the composite score as seen fit. If desired, additional columns containing pertinent information for the calculation of the composite score can be added to the global edges DataFrame and used in the composite score calculation. Once the composite score for each source node is calculated, values are stored in a Python dictionary that contains every source node’s unique identifier as a key and the respective composite score as the value. If the source nodes being displayed only have a single outgoing edge, the composite score is equal to this edge value.

The purpose of calculating the composite score within the graph object, as opposed to simply assigning composite scores to source nodes before the data is uploaded to CompositeView, is that manipulating the graph can change the composite score itself. For example, if a source node with three edge values has one of the edges filtered out of the graph, the composite score associated with that source node will change based on the new set of outgoing edges. The implication of this is that, depending on the data set, a user of CompositeView could easily filter edge values and trim target nodes to understand the impact of removing or adding back certain data. In the HDI example, filtering out one of the three metric values (modeled as target nodes) would result in a composite score that is not specifically equivalent to the HDI but might have some significance in its own right. Regarding SemNet, this can be crucial in understanding how certain biomedical concepts relate to the shared set of source nodes they are connected to. The composite score itself can be filtered in CompositeView, and the display size of the visualized source nodes is dependent on the composite score associated with them. Composite scores do not impact the size of target nodes, which are baseline larger than all source nodes for ease of identification.

#### 2.1.6. Graph Filtering and Interactivity

Visualizing with CompositeView allows users to be flexible with their data. An initially strict interpretation of the data can be made malleable with certain combinations of filters and changes, enabling insight that would have otherwise been missed. The interactivity is generally broken down into three categories: value filtering, node and type filtering, and graph manipulation.

Value filtering encompasses the ability to set bounds and custom limits to what nodes and edges are visualized. This is realized with three interactive components within CompositeView: combined value (composite score) filtering, edge value filtering, and max node count filtering. Each of these components allows the user to set a custom range in which the respective value can exist. To revisit the HDI example mentioned in Section 2.1.5, combined values can be filtered to only include countries that have a composite score between 0.5 and 0.75 (composite score is equivalent to the HDI value in this example); these countries lay somewhere in the upper-middle portion of the ranked HDI list.

Edge filtering allows the display of edge values to be controlled within a range. Under the assumption that edges share a similar scale and statistical nature, as understood in the definition of composite score, an interactive Dash sliding component allows the edge values shared between source and target nodes to be removed, and subsequently added back, based on their value. Edges that are filtered out will no longer contribute to the composite score, potentially changing both the size and meaning of a source node. As a final caveat involving filtering edge values, depending on how the composite score is calculated, sometimes edge value filtering can affect the global maximum or minimum combined value range. For example, take a graph with a global combined value range between two and eight, where each combined value is calculated by taking the arithmetic mean of outgoing source node edges. If a source node with outgoing edges evaluated at four, seven, and twelve has the lowest valued edge filtered out, its composite score is now outside the global graph bound. To combat this, the global combined value range adapts to the current state of the graph.

**Figure 6.**
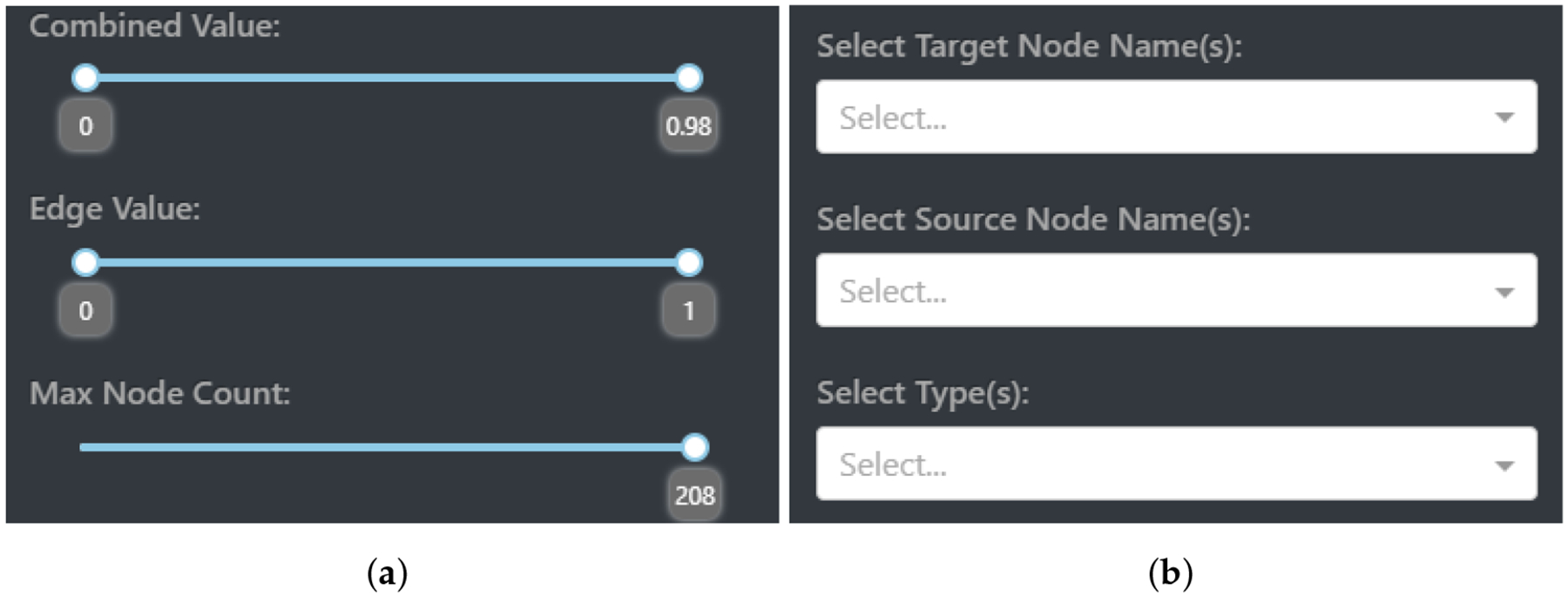
Value filtering as well as node and type filtering settings as displayed by CompositeView. (**a**) Value filtering sliders. (**b**) Node and edge filtering dropdowns.

The third, and potentially most straightforward, of the filtering components involves max node filtering. This component simply sets a ceiling for how many nodes can be visualized, determined by the composite score value. If the max node count is set to be half the entire number of source nodes, then the 50% of source nodes with a higher composite score than the lower 50% will be visualized. As a final note, filters are stacked in a specific way to allow for accurate interactions between one another. Edges are first filtered out, allowing new composite scores to be calculated based on resulting edges. Once these new composite scores are determined, they themselves are filtered. Finally, the max node count comes into play once both edge values and combined values are visited in turn. The value filtering interactive elements displayed by CompositeView can be seen in Figure 6a.

Node and type filtering is made possible by the use of Dash dropdowns, components that allow the user to choose values based on a predetermined set. In the case of target node filtering, this set consists of all the target nodes in the graph. The user can decide what target nodes to include in the visualization, which, in turn, can potentially impact what source nodes appear and how their composite scores are calculated. Take, again, the HDI example. If the education index target node is filtered out, then any source nodes exclusively connected to this target are also removed (which is none, in this case), and the composite score of the remaining source nodes is recalculated to not include the education index. This changes the overall meaning of the composite score assigned to each source node, a potential benefit to understanding the data. Filtering the source nodes performs a similar action with the same dropdown component, although this time the set of values provided to the user consists of all source node names.

Filtering the source nodes allows specific names to show up in the visualization, excluding any sources not explicitly specified. Filtering source nodes does not filter by source node ID, but rather by name itself, allowing multiple source nodes that share the same name (a common occurrence in analyzing multiple SemNet results) but different source node ID values to exist in the same visualization. Finally, filtering by type involves using all node type values as the input set for the third Dash dropdown component. The user can choose what node type is going to be displayed by CompositeView based on the options provided. As a last important note, when filtering for a specific source node type, target node type values must also be considered. Simply including source node type filters will display no nodes at all, since, without target node types (and therefore target nodes themselves) included, the source nodes by themselves have no edges and are filtered out, based on previously defined rules. The node and type filtering interactive elements displayed by CompositeView can be seen in Figure 6b.

**Figure 7.**
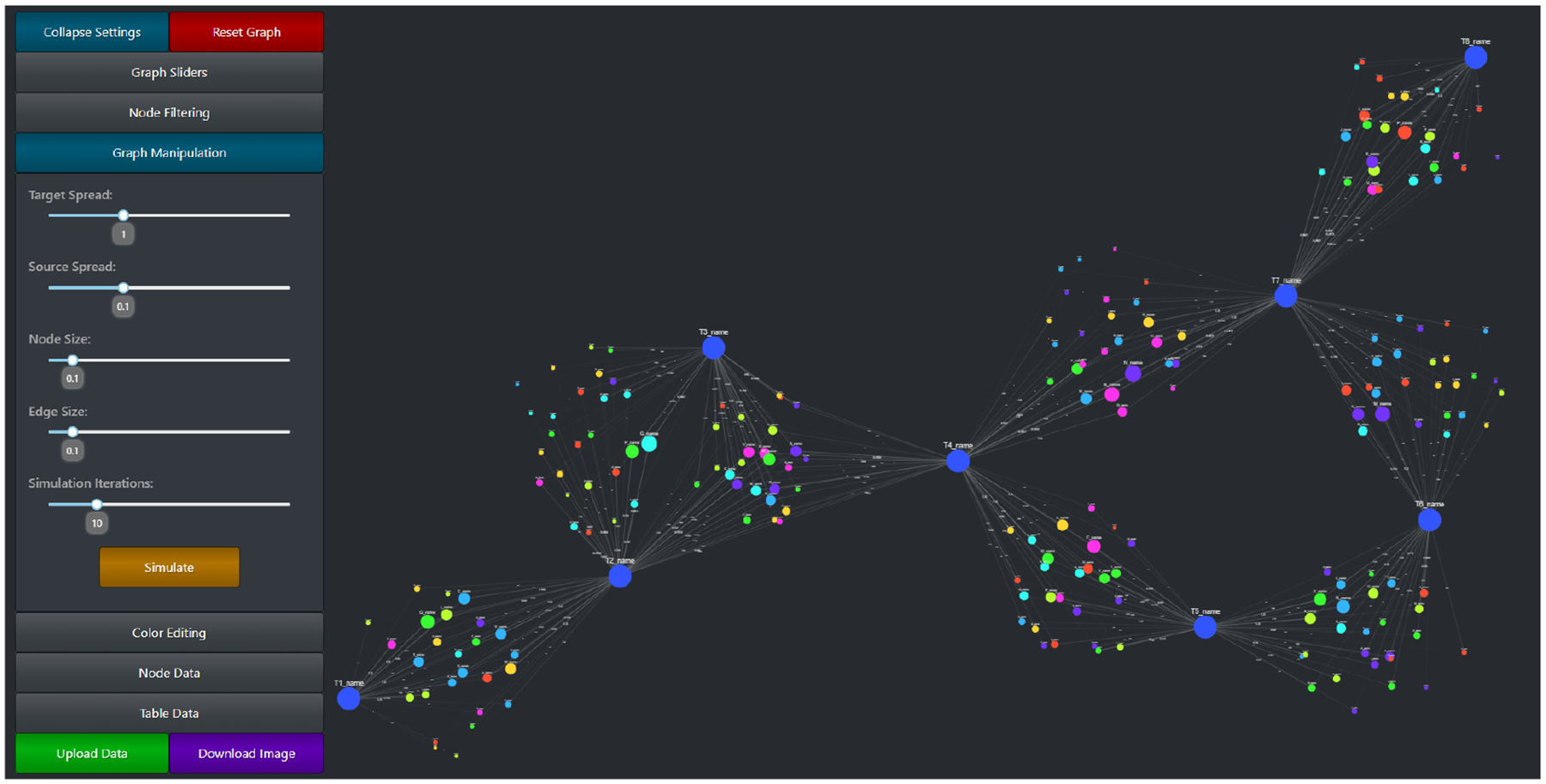
The complete CompositeView application layout (with Graph Manipulation settings open).

**Figure 8.**
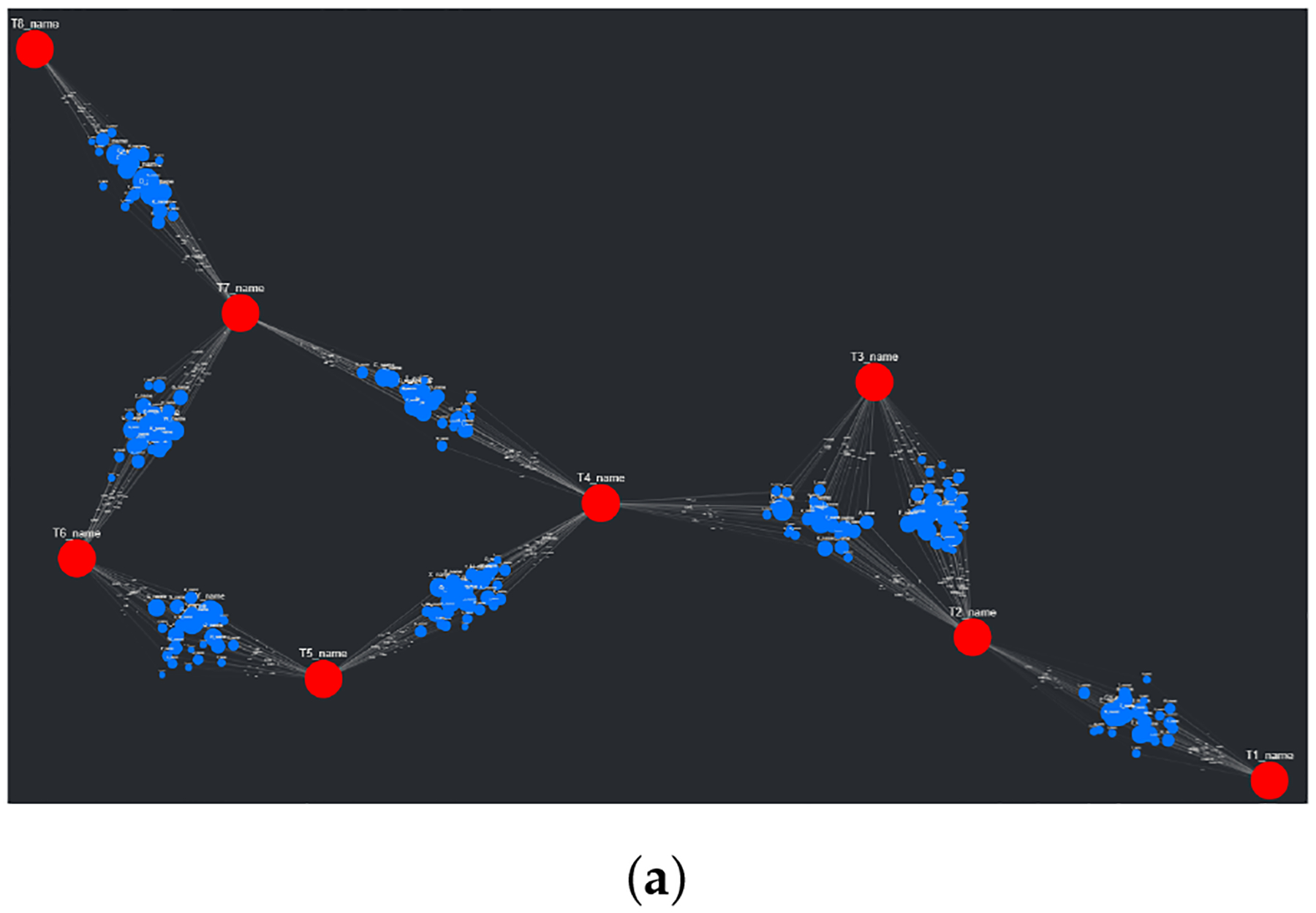

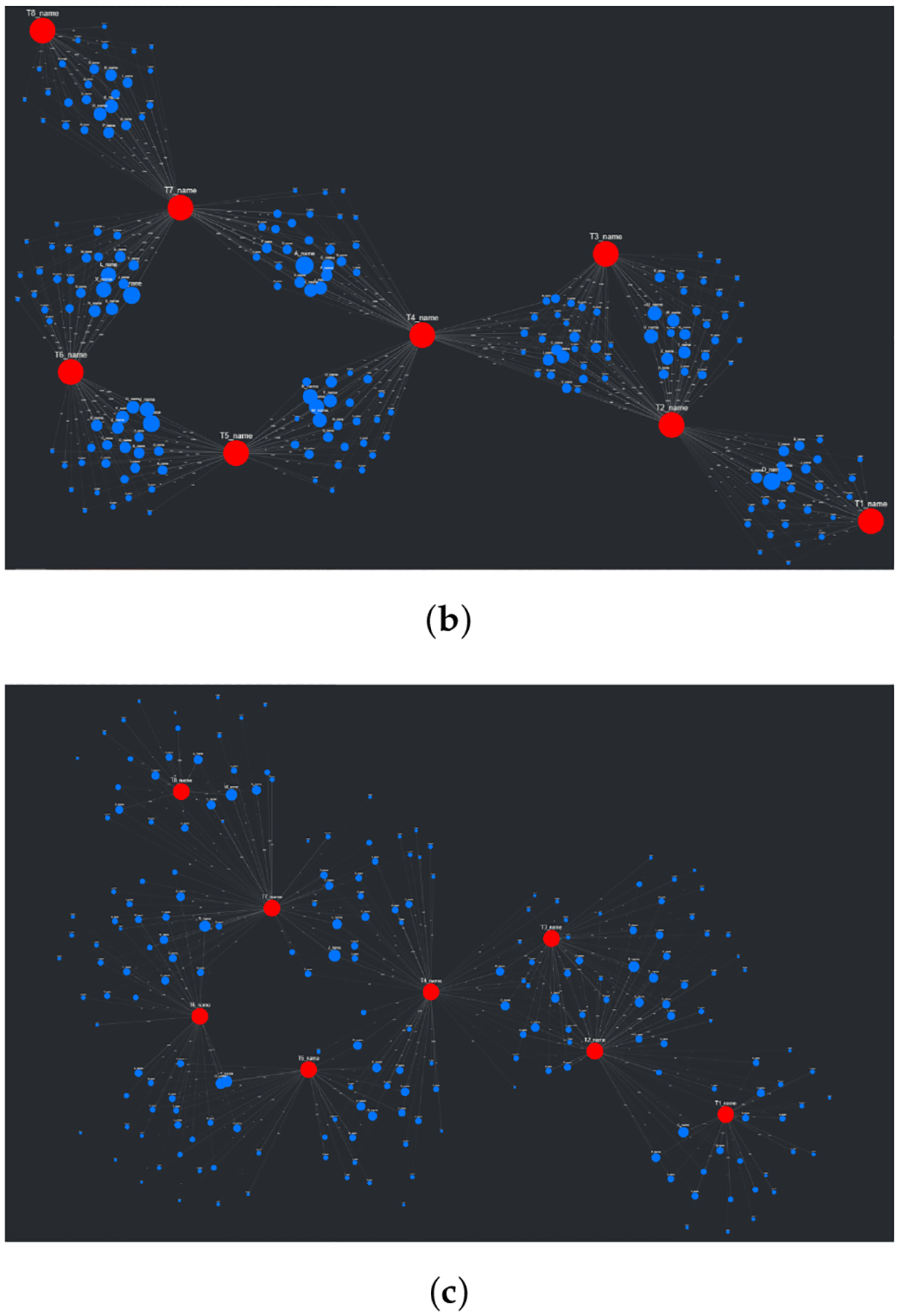
The impact of source spread (*k* in the NetworkX spring layout). The data shown is placeholder data used in CompositeView. (**a**) Source spread value half of base. (**b**) Base source spread value, the same as Figure 5c. (**c**) Source spread value double of base.

The graph manipulation settings, as well as the entire CompositeView application layout, can be seen in Figure 7. Graph manipulation entails both color editing and layout adjustments, along with a few other quality-of-life tools (such as node and edge size adjustment). Color editing provides color picking components that allow the user to specify source node color, source node gradient color, target node color, and selected type color (the type value of a currently selected node will be stored and used to determine a color change). Along with specific color editing components, a Dash button component is provided that randomizes both source and target node colors. Layout adjustment sliding components are the user’s tools to manipulate source and target node spread. Target spread influences the optimal distance between nodes (the *k* parameter in the NetworkX spring layout) for the first simulation in the adjusted spring layout method (Figure 5a). Increasing this value increases the “spread” of the initial target nodes. Source spread accomplishes a similar goal, but with only the source nodes that are populated after the target nodes are fixed. See Figure 8 to understand the impact of source spread; it is arguably the most important parameter impacting the readability of the graph. Depending on the data set, the source spread might need to be adjusted to make the graph baseline readable.

Two additional extremely important graph manipulation tools include the simulation iteration sliding component and the simulation button. The simulation iteration sliding component sets the number of iterations that the adjusted spring layout, specifically after the source nodes are added back, can run in the FR algorithm. Results shown in Section 3.1 shows the extent to which simulating the layout impacts the time it takes for the graph to completely finish rendering. By lowering the simulation iteration slider value, a trade-off between performance and aesthetics (and potential graph readability) is made. For large data sets, it might be prudent to initially set the simulation iteration value low and gradually increase it as nodes and edges are filtered out of the visualization. The simulation iteration value only impacts the simulation after the source nodes are added; before then, when target nodes are initially simulated, the performance impact is comparatively small. The simulation button itself simply re-simulates the graph. If a graph layout does not meet visualization standards, re-simulating it might help. The final graph manipulation components involve both node and edge size adjustments. These components exist only to help with visualization aesthetics. The ability to change both node and edge size allows for different sized graphs to be visualized using the same tool; a small graph could benefit from larger node and edge sizes, while a very large graph might benefit from smaller node and edge sizes (to prevent overlap).

### 2.2. Stress Testing Methods

To properly use CompositeView, understanding its scope and limitations is necessary. Recall that CompositeView has two logical groups: the application and the graph. Both of these components are individually timed. The application component is defined as all of the code that contributes to the creation of the application layout and callbacks. This includes generating callback decorators and functions, setting up Dash components, and defining layout styles and features. This does not include anything involving the Flask server, HTTP requests, data transfer between callbacks, or React.js rendering of the components and graph itself. This pipeline is well defined and very fast. Unless the rendered graph is intractably large (more than 5000 nodes typically), the impact of these elements is ultimately insignificant. The graph component consists of the Python class that is ultimately responsible for generating the NetworkX graph, updating the graph, specifying the graph layout, and outputting the graph elements list that is subsequently used by Dash Cytoscape.

The runtime will be recorded for both of these logical groups, with the graph timing split into two further components: graph initialization and graph update. The graph class itself is made up of many helper methods that act as building blocks that perform individual, yet harmonious, functions within CompositeView itself. In tandem, the methods involved with initializing and updating the graph utilize all of the “blocks” that are not computationally insignificant, while also providing an interpretable context for the timing results. The graph is initialized every time CompositeView starts or new data is uploaded (uploading data essentially reinstantiates the graph class). The graph is updated every time a filtering or graph manipulation interaction occurs. Generally, this update can either directly prune the elements list fed to Dash Cytoscape, or it can require the graph to be recreated and resimulated based on new parameters (this split is a result of runtime optimization and graph state caching). The former of these two situations is being timed, as the latter, for all intents and purposes, is largely the same as initialization but with new starting parameters.

The timing process begins with importing the Time module, provided by the Python Software Foundation. Next, a loop is created that iterates 500 times. During each iteration, data are generated, a new graph object is created and initialized, the application layout and callbacks are defined, and the graph is filtered and updated; all of these different actions simulate a typical use of CompositeView. The data generated during each iteration consist of a single target node connected to a number of source nodes determined by the loop variable. The number of source nodes generated per loop is 20(*loopvariable* + 1); the source node count starts at 20 and ends at 10,000. Each source node in these data is assigned an edge value of either 0.25 or 0.75 connecting it to the single target node, alternating after every iteration and resulting in half the edges being evaluated at 0.25 and the other half at 0.75. For each iteration, a new graph object is initialized and timed. All of the helper methods called when the graph object is initialized are also timed.

Once the new graph object is initialized, that same object is updated to filter out all source nodes with edge values below 0.5. In other words, exactly half of the source nodes are filtered out of the visualization. The time it takes for the graph to update, as well as the time it takes for all helper methods called during the update to run, will be recorded. Additionally, the time it takes to define the application layout, create the Dash callbacks, specify CSS and styling, and perform every other action not directly involved with the graph object and server, but still necessary for the application to run, will be timed. No aspect of building the application scales with the size of the data themselves, so the recorded time for each iteration is not expected to change. Finally, the time it takes for new graph objects to be created using previously defined attributes will be timed. Data transfer during application runtime is performed using JSON, a common data interchange format. Every time a callback is fired, all graph object attributes are stored in JSON and, subsequently, reapplied to a new graph object when required (essentially saving the graph object state). The time it takes the attributes to be loaded into the new object is what is being timed, as these attributes hold the graph data, which scale with graph size.

### 2.3. Preparing and Analyzing Sample Data

To showcase CompositeView’s power as a general-purpose visualization tool, three unique sample data sets have been chosen for visualization: network analysis data in the form of SemNet results, Human Development Index (HDI) data, and cardiovascular disease (CVD) data. Each of these data sets are cleaned and adjusted to fit CompositeView’s data input format detailed in Section 2.1.3. To further showcase CompositeView’s capabilities, each data visualization will be manipulated to gain new insight in a way that, without CompositeView, would be much more difficult. The raw data, as well as the Jupyter notebooks used to clean and format the data, are provided in the associated GitHub repository.

#### 2.3.1. SemNet Data

The first sample data set, the SemNet results data, is in actuality two data sets combined into one. These two data sets consist of the results of two individual SemNet runs using the target nodes Alzheimer’s disease (AD) and hypothyroidism. All intersecting nodes of one path length away from each target (within the SemNet knowledge graph) that are categorized under the Unified Medical Language System (UMLS) semantic type categories “Amino Acid, Peptide, or Protein” and “Disease or Syndrome” are used as the shared source node set. This set of source nodes is then ranked in respect to both targets using the exact mean HeteSim algorithm with a specified metapath length of 2. Both uses of SemNet produce a pandas DataFrame consisting of six columns: “source_node”, which contains the CUI identifiers for each source node in the shared set, “source_name”, which contains the name for each source node in the shared set, “source_type”, which indicates the source node’s UMLS semantic type, “target_node”, which contains the CUI identifier for either AD or hypothyroidism, respectively, “target_name”, which contains the name of the respective target node, and finally “hetesim_score”, which is the aggregate HeteSim score associated with each source–target pair. The SemNet output is (by design) formatted similarly to Table 3, so combining the two SemNet results DataFrames into a single DataFrame to meet the input data format requirements is straightforward. The column “target_type” specified in the input data format table is populated with “target_node” to uniquely categorize each node in the eventual visualization. Once the combined DataFrame is generated, it is exported as a CSV file and subsequently uploaded to CompositeView.

Once the formatted CSV file is uploaded to CompositeView, the visualization is manipulated to filter out edges above or below the inner 50–75 percentile range. This specific manipulation is particularly useful since, in SemNet’s current knowledge graph, there is a prevalence of highly connected, generic nodes (nodes representing concepts such as “water” or “protein” that hold little significance). As sources, these generic nodes are often assigned very high aggregate HeteSim scores which CompositeView can filter out of the graph. Adjusting the edge value range can allow for a “sweet spot” to form for SemNet results, where source nodes that are highly ranked, but not too highly ranked, are visualized and distinguished. The resulting visualizations, both unadjusted and adjusted, are described in Section 3.2.1.

#### 2.3.2. Human Development Index Data

The second of the three sample data sets consists of Human Development Index (HDI) data taken from the 2020 Human Development Report [10]. These data, similar to the SemNet sample data, are actually a combination of four data sets, where 189 countries are evaluated based on life expectancy at birth, expected years of schooling, mean years of schooling, and gross national income per capita. Each of these four metrics is normalized (then combined, where required) between zero and one, resulting in the three resulting indices: the life expectancy index (life expectancy at birth, normalized), the education index (both expected years of schooling and mean years of schooling are normalized, then the arithmetic mean is taken), and the income index (gross national income per capita, normalized). Each of these three index values is modeled as a target node, each of the respective countries is modeled as a source node, and the actual index value shared between each country and the respective index is modeled as each source–target pair’s edge weight. Each country in the final formatted input data is assigned a group, ranging from “low development” to “high development”. These categories are determined by the HDI value assigned to a country, which is calculated beforehand and then disregarded to better showcase CompositeView’s capabilities. Once the HDI data are formatted based on the specifications detailed in Section 2.1.3, they are exported as a CSV file and subsequently uploaded to CompositeView.

After the formatted CSV is uploaded to CompositeView, the target node “education_index” is filtered out of the visualization. This leads to the composite score associated with each source node (country) to no longer include the education index value in the geometric mean, resulting in the composite value itself no longer being equivalent to the HDI value. This new composite score, though no longer labeled as the HDI value, holds its own significance in a certain context. After “education_index” is filtered out, the maximum number of displayed nodes is adjusted to allow only the top three nodes by composite score to be visualized. The resulting visualizations, both unadjusted and adjusted, are described in Section 3.2.2.

#### 2.3.3. Cardiovascular Disease Data

The last of the three sample data sets involves cardiovascular disease (CVD) 10-year risk assessment data, originally taken as a sample from the Isfahan Cohort Study (ICS). These data were originally used to help determine a new risk assessment chart for CVD using the PARS model, a novel method for predicting a patient’s 10-year risk of CVD occurring [11]. These data, in the context of the PARS model and risk assessment charts, are an example of data that are not originally well suited for CompositeView. The risk factors, which would be modeled as the target nodes, are often measured using discrete values (such as sex, presence of smoking, etc.). These discrete values, along with continuously measured risk factors such as blood pressure and cholesterol concentrations, do not exist on the same scale and, therefore, the edge value slider used in CompositeView has little practical use. Part of the Cox model used to compute the 10-year risk of CVD involves scaling the difference between respective mean risk factors and measured risk factors by the hazard ratio. These resulting values could potentially be the associated edge values used by CompositeView (as they are ultimately summed to help generate the 10-year risk probability); however, the efficacy of this has not been determined. Instead, these data are used by a different model developed in D’agostino et al. [47] (an inspiration and comparison study used in the development of the PARS model) to generate values that can be used as edge weights in CompositeView. This new CVD 10-year risk assessment algorithm, known as the Framingham Risk Score (FRS), is used to map risk factor measurements to point values, which are subsequently summed to generate a composite score. This composite score determines the overall risk of a 10-year CVD event occurring. The risk factors used by the FRS, as described in D’Agostino et al, do not perfectly overlap with the ICS sample data risk factors; some adjustments have therefore been made. The efficacy of these adjustments is ultimately inconsequential (though intuitive), as the data and algorithms are used to explain CompositeView. All adjustments are nevertheless documented in the Jupyter notebooks within the GitHub repository attached to this paper.

Ultimately, the risk factors used in the Framingham Risk Score are modeled as target nodes, the patients themselves are modeled as source nodes, and the risk factor measurements, converted to point values per the FRS model, are modeled as each respective source–target pair’s edge weight. Additionally, each source node is categorized by sex; the Framingham Risk Score charts are delineated based on sex, making this particular categorization intuitive for the eventual visualization. Once the raw data are cleaned and formatted per Section 2.1.3, they are exported as a CSV and subsequently uploaded to CompositeView. As a test analysis, all but the two target nodes “age” and “smoking_status” are filtered out of the visualization. D’Agostino el al. notes that these risk factors are especially impactful in the likelihood of a CVD event occurring within 10 years, as they are assigned particularly high point values, especially age. The resulting visualizations, both unadjusted and adjusted, are described in Section 3.2.3.

## 3. Results

### 3.1. Stress Testing Results

**Figure 9.**
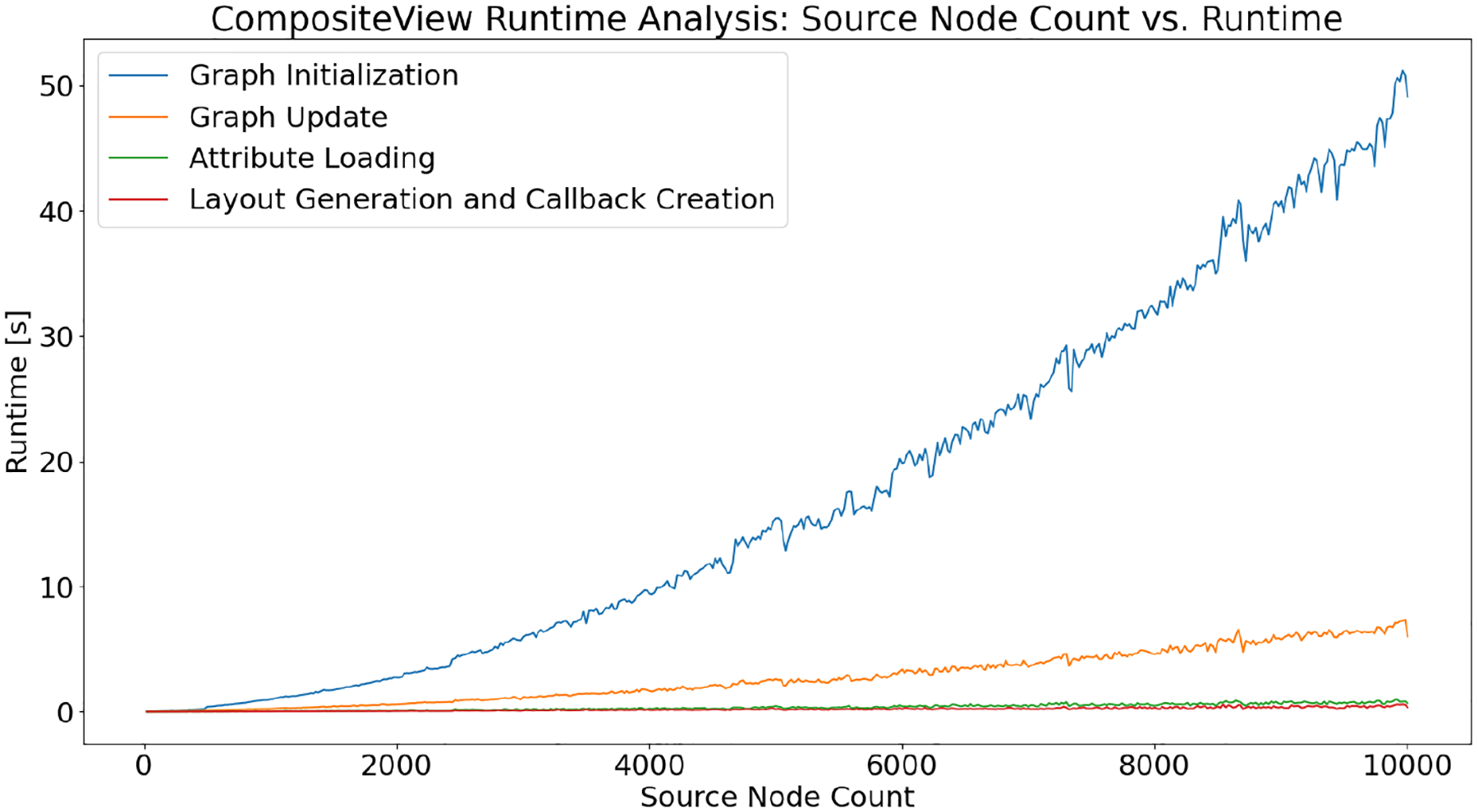
Application startup, graph initialization, attribute loading, and graph update.

**Figure 10.**
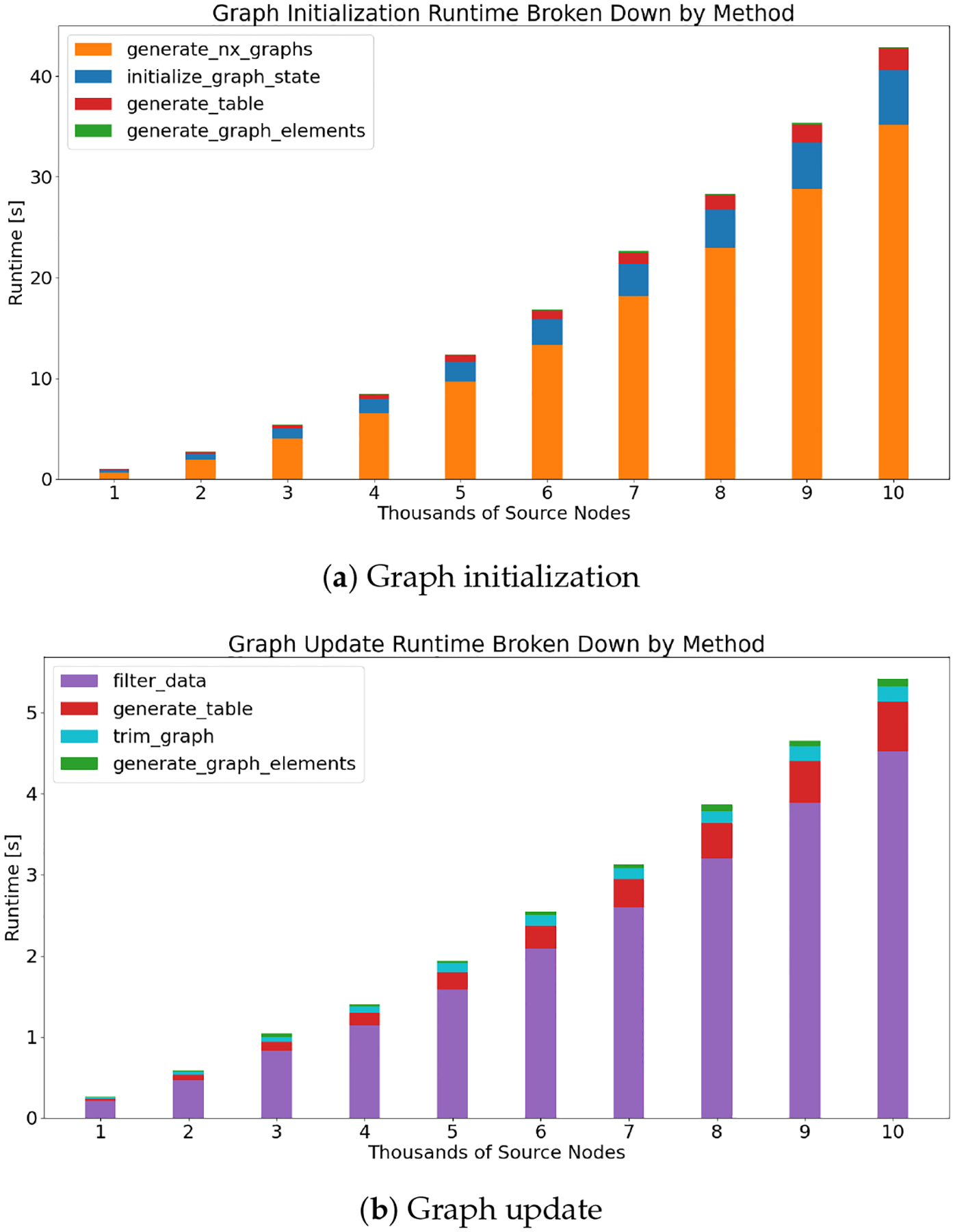
Runtime analysis of graph startup and update, broken down by most important methods.

The timing results for the process described in Section 2.2 can be observed in Figures 9 and 10a. Figure 9 visualizes the timing impact of increasing the number of source nodes and edges on graph initialization, graph update, attribute loading, and application layout and callback creation. Graph initialization makes up the majority of runtime observed in this initial analysis, which is unsurprising given that simulating the graph layout, the most computational taxing component of the visualizer, is performed in full when the graph object is first instantiated. The breakdown of the most impactful methods within the graph initialization can be seen in Figure 10a. Not all methods used during initialization are included. A select few, such as those tasked with creating the pandas DataFrame or holding the edge data or color mapping, are computationally inconsequential.

Updating the graph is the second most time-consuming process, as seen in Figure 9. When nodes and edges are removed from the graph, in manner similar to how half of the source nodes are removed from the visualization, as described in Section 2.2, the elements list that is read by Dash Cytoscape is directly modified. Each node that is filtered out is removed from this list, eliminating the need to resimulate the layout, the most computationally expensive part of the Visualizer. Filtering the data, the process of adjusting the core pandas DataFrame of graph edges based on user input, is the most computationally expensive part of updating the graph, assuming the update does not involve simulating the graph layout. When the graph layout is resimulated, nodes that have been filtered out no longer hold position information. When these nodes are reintroduced into the graph, a new layout must be simulated to assign position information to these newly added nodes. When removing nodes from a graph that has already been simulated, the position information for that individual layout simulation is stored and can be reintroduced if that node is added back into the visualization. This process was designed to cache graph states and reduce the need to simulate graph layouts, given that this process is far and away the most taxing on runtime. Beyond initializing and updating the graph, both attribute loading and application layout and callback creation are computationally inconsequential, even when the data used by the graph are increasing in number. As a final note, once the graph layout is rendered using Dash Cytoscape, depending on the total number of nodes and edges being visualized, there is some “sluggishness” associated with an increased number of nodes. This is a difficult thing to measure. Ultimately, reducing the overall node count through filters or changes to initial graph parameters can improve performance immensely and is suggested.

### 3.2. Visualizing Sample Data and Analysis Results

#### 3.2.1. SemNet Data

**Figure 11.**
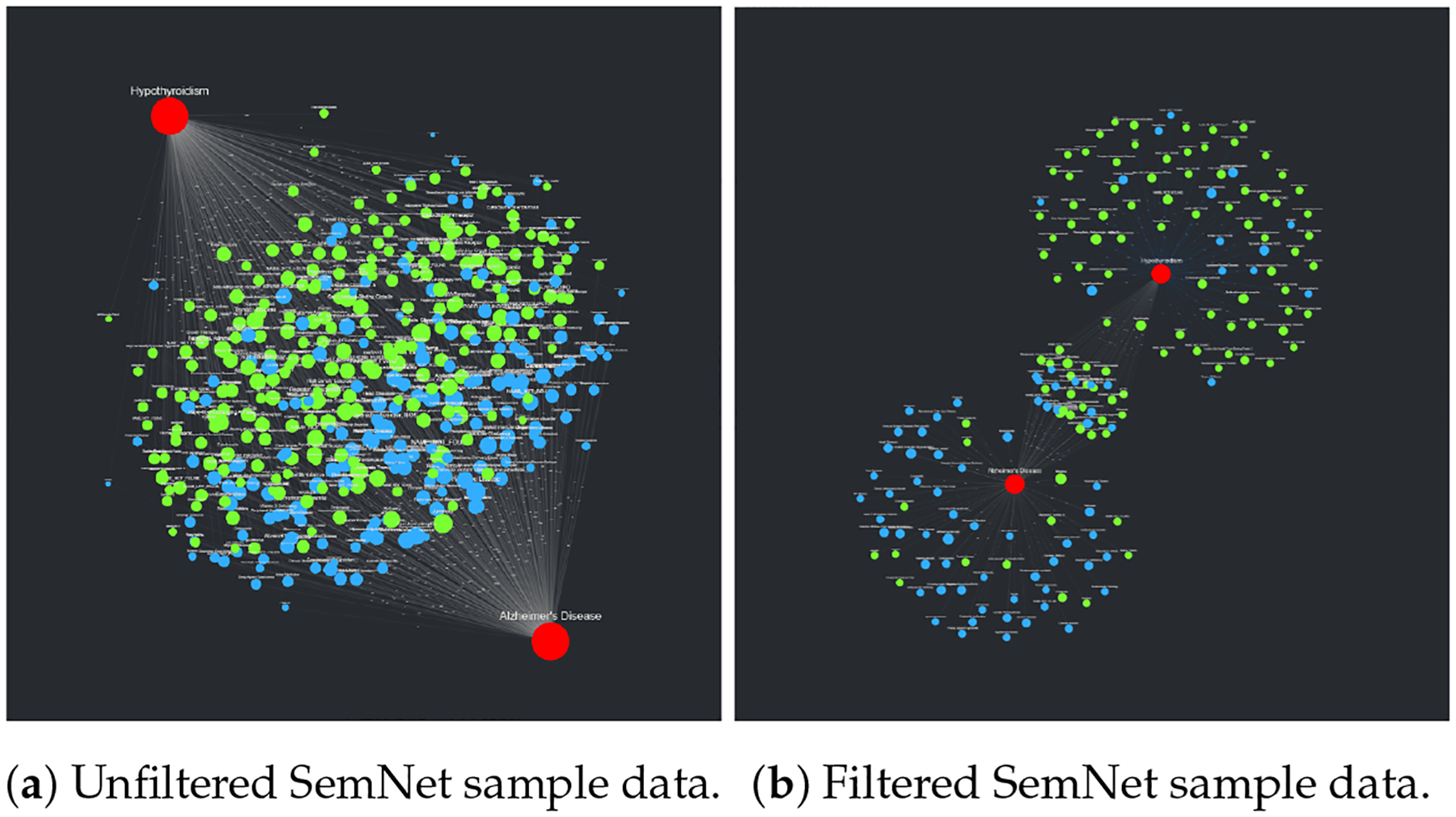
The SemNet sample data, both unfiltered (**a**) and filtered (**b**), based on criteria described in Section 2.3.1.

The sample SemNet data described in Section 2.3.1 were uploaded to CompositeView as a CSV file; the resulting unfiltered visualization can be seen in Figure 11a. In this visualization, nodes are colored based on their associated types or groups: red indicates target nodes, blue indicates UMLS semantic type “Disease or Syndrome”, and green indicates UMLS semantic type “Amino Acid, Peptide, or Protein”. After the data was filtered per Section 2.3.1, the visualization seen in Figure 11b was generated. There are three distinct clusters of source nodes: two surrounding each target node, and one shared between both target nodes. Recall that the filtering applied to this data limits edge values to the 50–75 percentile range; this explains the source nodes with only one edge connected to one of the two respective targets. The cluster of source nodes shared between both targets represents the “sweet spot” where each node is highly related to both Alzheimer’s disease and hypothyroidism per the HeteSim relevance measure within the SemNet knowledge graph.

#### 3.2.2. Human Development Index Data

**Figure 12.**
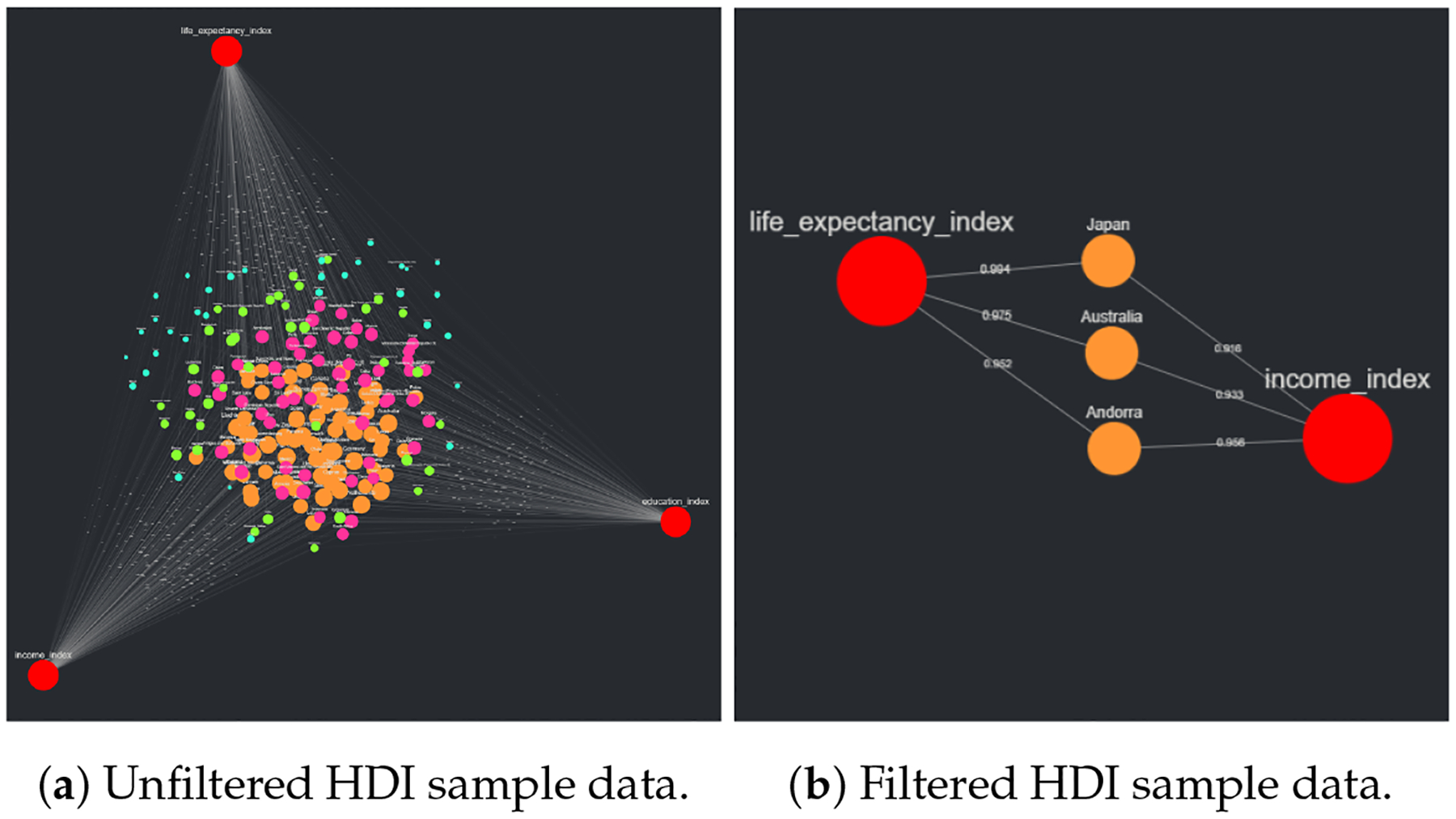
The HDI sample data, both unfiltered (**a**) and filtered (**b**), based on criteria described in Section 2.3.2.

The formatted HDI data as described in Section 2.3.2 was also uploaded to Composite-View as a CSV; the resulting unfiltered visualization can be seen in Figure 12a. Source nodes are colored based on development category, with each color mapped as follows: orange is “very high development”, pink is “high development”, green is “medium development”, and blue is “low development”. Target nodes, as in the sample SemNet data visualization, are colored red for ease of identification. After the data were filtered per Section 2.3.2, the three resulting source nodes were Australia, Japan, and Andorra. With the absence of the “education_index” target node, these three source nodes had the highest composite score. The filtered visualization is shown in Figure 12b. The three highest ranked source nodes with all three targets visualized (the composite score then being equal to the HDI value) were Norway, Ireland, and Switzerland, implying that these countries rely on the education index to reach their high HDI scores. As a final point of validation, the top ten composite scores with all three targets included in the visualization (and therefore composite score) correspond exactly with the HDI ranking and respective values.

#### 3.2.3. Cardiovascular Disease Data

**Figure 13.**
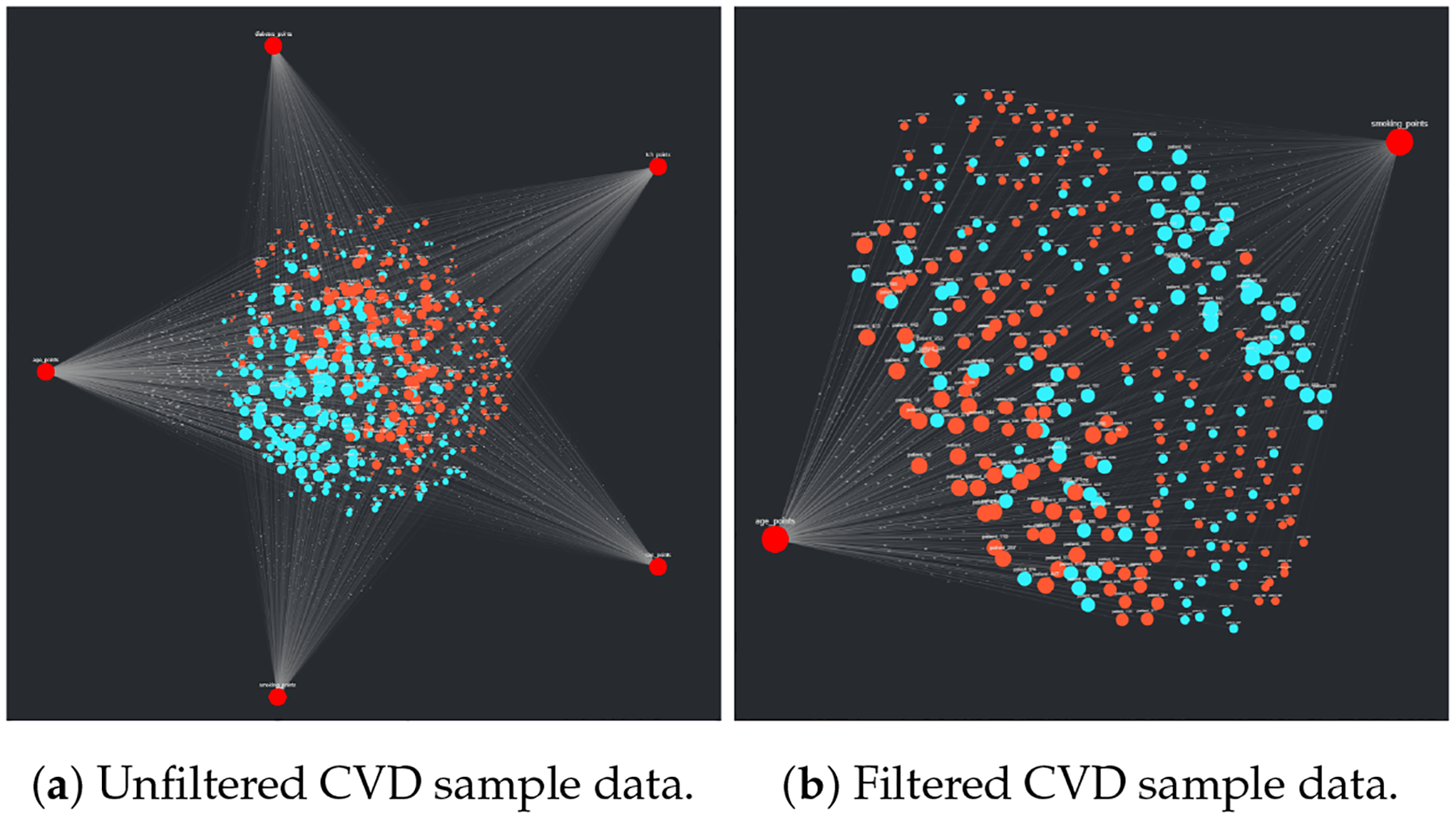
The CVD sample data, both unfiltered (**a**) and filtered (**b**), based on criteria described in Section 2.3.3.

The final of the three sample data sets, the CVD 10-year risk assessment data, was uploaded to CompositeView as a CSV; the resulting unfiltered visualization can be observed in Figure 13a. Like the previous two sample data visualizations, the target nodes are colored red for ease of identification. The source nodes are categorized by sex and colored accordingly: blue indicates male, orange indicates female. After the data was filtered per Section 2.3.3, the resulting visualization seen in Figure 13b was displayed. The larger source nodes indicate “higher risk” of a 10-year CVD event occurring. Interestingly, the higher risk male source nodes tend to gravitate towards the “smoking_status” target node and the higher risk female source nodes tend to gravitate towards the “age” target node. The implication is that male patients are more likely to be higher risk due to smoking than female patients.

## 4. Discussion

### 4.1. Related Work and Comparisons

Data visualization is certainly not a novel concept. There are many data visualization tools (or tools that can visualize data, even if their principal design intent was not specific to data visualization). Here, we compare CompositeView to six relatively common data visualization tools ranging from “least” to “most” similar to CompositeView: Excel, Tableau, Cytoscape, Neo4j, NodeXL, and Gephi. Excel and Tableau are general data visualization tools which are not specific to network visualization. Cytoscape is an open-source bioinformatics software platform for visualizing molecular interaction networks and integrating with gene expression profiles and other state data. Neo4j is primarily an open-source graph database software with some graph visualization ability. NodeXL (or the paid upgrade, NodeXL Pro) is a social network analysis and visualization tool. Finally, Gephi is a general network visualization and analysis tool which is not customized to a specific data domain.

Unlike CompositeView, none of these comparison tools have the ability to automatically calculate composite scores “on the fly.” With significant coding by the user, some other visualization tools (Excel, Tableau, Cytoscape, and Gephi) could be adapted to calculate network composite scores, but the composite scores will not automatically update when changing the graph layout or doing filtering customization. This is the principal difference between CompositeView and all of the tools qualitatively compared below. CompositeView is not simply a network visualization tool; rather, it is a tool to calculate composite scores that interactively update as the user zooms in or out of a graph to examine composite relationships across varying levels of data aggregation. Live, automatic updating of composite scores while interacting with or filtering data is key for a domain user to more efficiently decipher actionable insight from complex relationships.

In summary, CompositeView fills a niche gap compared to other currently available tools by: (1) automatically calculating and automatically updating composite scores as the user interacts with the graph to filter or aggregate data; (2) providing layouts and features that optimally decrease information overload for the specified data format; (3) performing interactive composite scoring not only for network data, but also for non-network data, which enables actionable insight to be deduced from a wider variety of large, complex data sets.

#### 4.1.1. Comparison to Excel

Excel is a software produced by Microsoft (Redmond, WA, USA; www.microsoft.com) that enables the analysis of spreadsheets and tables, its primary data structure. These data, which optimally range from small to medium in size, can be quickly and easily analyzed using an assortment of Excel functions. Excel has advantages over CompositeView in many respects, particularly in its general-purpose usability and optimization; however, in the specific realm of composite score data, as described in Section 2.1.3, CompositeView has its own advantages. Excel lacks the network visualization power of Cytoscape as well as the precise, customizable scripting capabilities of Python, two core advantages of CompositeView. Additionally, the interactivity provided by Dash and its assortment of components and callback functions can, in many ways, outperform Excel in this context. Finally, the ability to easily distribute CompositeView (in the form of web application hosting) is arguably higher than Excel. Ultimately, certain network analysis data and more general composite score data lend themselves very well to network-based visualization and interactivity, which favor CompositeView.

#### 4.1.2. Comparison to Tableau

Tableau [4] is an extremely powerful data visualization software that specializes in ease of use and broad applicability. Its drag and drop design, paired with custom calculation fields and filters, makes it intuitive and well suited for many types of visualizations, specifically dashboards. Tableau shares many potential drawbacks with Excel, however, particularly in network-based visualization and flexibility. What might seem counter-intuitive at first is that CompositeView’s relatively narrow scope can, in many ways, improve its flexibility in respect to other visualization tools. To clarify, much of the calculation functionality in Tableau can be quite restrictive in support of some of its core design choices such as ease of use and broad applicability. This calculation limitation (something not as pronounced in Excel), along with a relatively rigid framework, make Tableau great for fast, practical visualizations but potentially not as great for visualizing composite score data in an interactive way, particularly when modeled as a network. As a general theme, CompositeView provides fast, flexible, and easy visualization capabilities for a specific data format that would otherwise be more difficult to visualize and interact with using other tools that are designed to be more general.

#### 4.1.3. Comparison to Cytoscape

Cytoscape [39] is an open-source software platform for both visualizing and analyzing complex networks and integrating them with attribute data. Originally built to visualize biomolecular interaction networks, Cytoscape has since expanded to include a sister project, Cytoscape.js, to generalize the platform and allow in-browser visualizations that support a variety of plugins. In fact, one of the key tools CompositeView uses is Dash Cytoscape, a translation of Cytoscape.js features that are integrated into the Dash framework. What, therefore, is the point of CompositeView if Cytoscape ultimately exists? In essence, CompositeView does not visualize networks: it utilizes networks to visualize composite score data. The elements used to filter, manipulate, color, and effectively simulate the data have already been built into CompositeView; as long as the input data format is met, and the data align with the “composite score” definition, CompositeView is very simple and quick to use. Visualizing similar data using Cytoscape alone would constitute building custom filters and sliders, calculating the composite score manually, and learning a potentially complex tool for a very specific use case. As a final comparison, CompositeView is built entirely in Python (using specifically chosen packages to ensure this unilateral theme) to allow for easy integration with other Python-based data analysis pipelines. Given Python’s popularity, this was a deliberate design choice.

#### 4.1.4. Comparison to Neo4j

Neo4j (https://neo4j.com) is a graph database software with native graph storage and processing but primarily external visualization. It is most known for its database management system rather than its visualization features. Neo4j utilizes additional embeddable tools (neovis.js, popoto.js), embeddable connections, embeddable libraries (d3.js, vis.js, sigma.js, vivagraph.js, or cytoscape.js), or stand alone products (neo4j Bloom, Tableau, Linkurious, or Keylines, etc.) to perform data visualization of data stored in the neo4j graph database. However, the embeddable open-source Cytoscape library is one of the most popular and primary embeddable libraries used to perform data visualization. Cytoscape was described in detail above and also forms part of the foundation of CompositeView. While neo4j is convenient for querying a database, it is known to be sluggish when performing multiple queries on a large graph. For example, the replacement of neo4j with nested Python libraries resulted in multiple orders of magnitude of speed up in the SemNet 2.0 biomedical knowledge graph analysis software [9]. Given that neo4j is mostly reliant on other embedded tools or external products, it does not have the built-in functionality to update composite scores on the fly as a user interacts with the data to examine varying levels of filtering or aggregation.

#### 4.1.5. Comparison to NodeXL

NodeXL (https://nodexl.com) is a true network data visualization tool that utilizes the Microsoft Excel environment. Essentially, NodeXL is an add-in to Microsoft Excel. It can take input lists of nodes and edges to illustrate relationships in the network. NodeXL was primarily developed for social network analysis, as evidenced by its advertised connection to the Social Media Research Foundation as of the time of the time of publication. NodeXL comes in a free version as well as NodeXL Pro, which is a paid service. The open-source NodeXL version enables basic user features such as: shape, color, size, opacity, and label of vertices; calculation of overall network metrics such as density or modularity; and calculation of basic vertex metrics such as degree, in-degree, or out-degree. The NodeXL Pro version unlocks more analytical capability and convenience features, including advanced network metrics (betweenness centrality, Eigenvector centrality, etc.), content analysis (text sentiment, time series, hashtag counts), social network API(s), data import/export, and some automation. The ability to interact seamlessly with Excel is one strength of NodeXL or NodeXL Pro; many data sets are naturally stored in CSV, XLS, or XLSX files, which are native to Excel. However, the largest strength of NodeXL and especially NodeXL Pro are convenience features that make it especially suited for social network analysis. However, neither NodeXL nor NodeXL Pro has built-in interactive calculation of composite score data in the manner of CompositeView. Additionally, NodeXL is meant to handle smaller graphs (in terms of number of nodes and edges) compared to CompositeView or Gephi, the latter of which is discussed below. As such, NodeXL cannot replace the utility or function of CompositeView for composite scoring visualizations.

#### 4.1.6. Comparison to Gephi

Gephi [5], like Cytoscape, is an open-source software platform for both visualizing and analyzing complex networks. Gephi differs from CompositeView, Cytoscape, and NodeXL in its use case as a more generalist network visualization tool. As a pure network visualization tool, Gephi has many great features. It allows the user to choose multiple layout options, color coding patterns, and filters that are more granular and customizable when compared to options provided by NodeXL or CompositeView. CompositeView, however, is not built as a dedicated network visualization tool, but rather as a tool that utilizes network visualization techniques to display and interact with composite score data. CompositeView automatically calculates composite scores for source nodes (assuming the data complies with the format described in Section 2.1.3) and allows the user to filter source nodes, target nodes, and their associated values dynamically based on this calculated score. Gephi can also embed attribute data, such as a composite score, but it must be calculated beforehand and assigned to nodes individually (a difficult task, especially if the user has minimal programming experience). Additionally, this composite score does not change with filtering actions. Finally, the use of Dash with CompositeView allows a user with programming experience to adjust the application and create a more flexible dashboard beyond just a network visualization tool. Gephi is a dedicated network visualization tool, while CompositeView can be more flexible.

**Figure 14.**
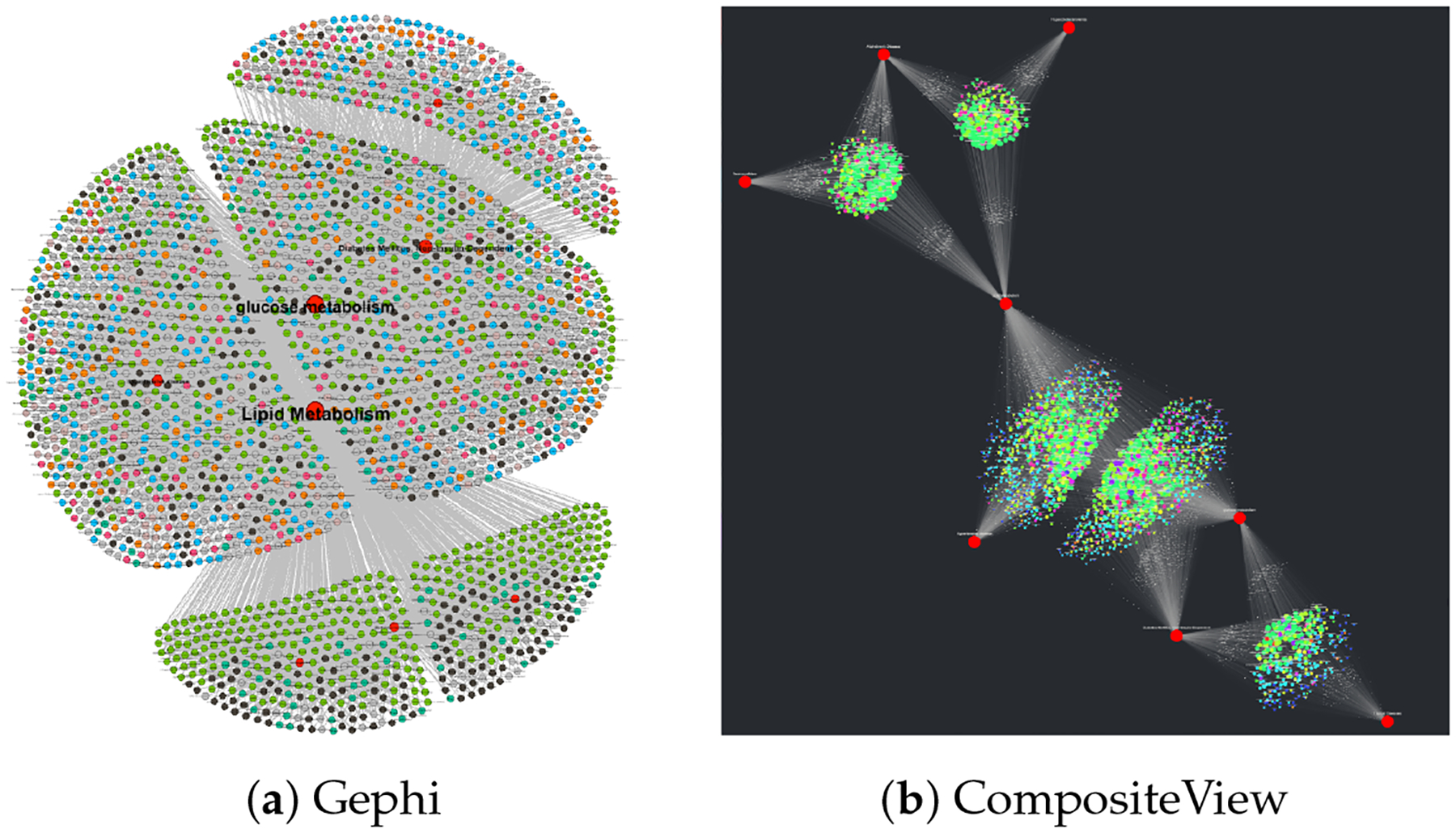
Visual comparison between Gephi and CompositeView using the same SemNet data set seen in Figures 3 and 4 (approximately 2472 source nodes). The red circles represent the main input features or nodes for which relationships are being visualized. In this example, visualized relationships for composite data are much easier to deduce with CompositeView compared to Gephi.

Figure 14 displays the same SemNet data using the two tools, Gephi and CompositeView. CompositeView utilizes the adjusted spring layout, as described in Section 2.1.4, while Gephi, a more generalist tool, utilizes the Fruchterman–Reingold algorithm, which results in a layout with ambiguously assigned source node clusters. This example illustrates that even the static visualization is much improved with CompositeView over Gephi. It is much easier for CompositeView to visually discern key relationships between defined target nodes and groups of related source nodes. However, the biggest difference is that the Gephi-produced image cannot be dynamically updated with new composite scores computed after filtering, adjusting node counts, or adjusting any other displayed feature. By contrast, CompositeView can dynamically update the visualized image as the user crafts a visualization best suited for acquiring insight. Thus, while Gephi is the closest to CompositeView in terms of its purpose, function, and features, Gephi still cannot replace the function or utility of CompositeView.

### 4.2. Limitations

First, CompositeView is ultimately designed to visualize data of a specific format proposed in Section 2.1.3, which closely resembles SemNet or SemNet 2.0 results. Three test cases were utilized to stress test CompositeView’s flexibility during development. For example, test data sets with high-precision edge values produced problems in early development which have since been resolved.

Second, CompositeView was developed using Windows 10 and Google Chrome, both of which are common but not ubiquitous. Compatibility testing with Linux, macOS, and a myriad of browsers has been conducted successfully, and CompositeView’s performance across these different environments is noted in its documentation. Ultimately, Dash is the tool that runs the application and determines its compatibility with different browser and operating system setups. To better understand problems specific to any browser or operating system, please refer to Dash documentation [48].

Third, CompositeView relies on a multitude of open-source Python packages to generate the visualization. A reliance on these packages can potentially open up a black box of errors that cannot be easily resolved. These packages have been very specifically chosen for their compatibility and ease of use, but their versions should be the same as those indicated in CompositeView’s documentation (though different package versions are usually acceptable, given that they are also compatible with the other packages included). It is recommended to run CompositeView using a virtual environment, if it is not being externally hosted.

Fourth, CompositeView, in its current state, requires a very small, but non-zero, amount of Python knowledge to run locally. For the web application to start, the Python script that specifies the layout, callbacks, elements, and other Dash components needs to be run. In order to use CompositeView without any prerequisite Python knowledge, the Dash application that forms the backbone of CompositeView must be hosted using either cloud-based services or a personal machine (for others to externally use). Dash documentation exists that describes possible avenues for application hosting [49]; this has proven to be an easy and effective way to completely remove the necessity of Python experience for CompositeView users.

### 4.3. Applications and Future Direction

CompositeView was motivated by and originally developed for examining knowledge graph relationship ranking results, such as those produced by both SemNet version 1 [8] and SemNet version 2 [9]. As such, CompositeView enables SemNet users to gain deep insight from the results in a fraction of the time previously spent parsing through tables. As previously noted, CompositeView is not limited to SemNet-related data, and the tool’s advantages can be translated to other similar network analysis algorithms and pipelines.

#### 4.3.1. Bioinformatics and Network Analysis Applications

Bioinformatics, a field closely adjacent to computational biology, is the science of storing, disseminating, and analyzing biological data. These data usually come in the form of DNA and amino acid sequences, and bioinformaticists have been able to conduct in-depth studies of gene-gene, protein–protein, and gene–protein relationships using network analysis. This leads to a more comprehensive example of a potential application of CompositeView, in addition to those mentioned in Section 1.2: inFRank, a ranking-based method for identifying influential genes [50]. Described in Cui et al., the inFRank analysis method consists of three steps: the creation of a background network, the modification of this network using biological process-specific data, and, finally, influence score calculations using inFRank itself. For the main experiment described in Cui et al., the background network was initially developed using data from KEGG pathways and protein–protein interaction networks described in Rolland et al. [51], and it was subsequently modified by weighting edges based on gene expression data, specifically hepatocellular carcinoma (HCC) RNA-seq data. Once the network was created, inFRank was used to rank the top 20 most influential genes in relation to HCC, with further investigation leading to a potential therapeutic area in the form of five critical genes responsible for mitotic spindle assembly checkpoint.

The results of the HCC experiment alone could benefit from CompositeView, where each gene is modeled as a source node, HCC is modeled as a target node, and the inFRank influence score is the weighted edges; however, Cui et al. also describes a pan-cancer analysis where gene influence scores for 18 different cancers identified the top 20 influential genes for each cancer. Many of the influential genes overlap. This pan-cancer analysis is a perfect example of practical network analysis that could benefit from CompositeView. Similar to the HCC experiment, each cancer type could be modeled as a target node, each gene could be modeled as a source node, and the inFRank similarity value could be the assigned edge weight. The genes that are highly influential for multiple cancers would have an associated composite score that reflects, in some way, their joint influence. Additionally, cancers originating from the same or closely related organs or tissues could be grouped together, and genes that tend to influence the same set of cancers could also be grouped together. The addition of CompositeView to similar applications, even those not strictly using inFRank (though performing similar analyses), could be extremely beneficial.

#### 4.3.2. Assessment of Decision Tree, Bayesian Network, and Neural Network Applications

Another area where CompositeView could prove useful is machine learning, specifically by enhancing the interpretability of common machine learning and statistical analysis techniques, network-related or otherwise. CompositeView could be used to look at larger forests and their indices for determining decision splits or their voting patterns [52]. Additionally, CompositeView could be used for visualizing Bayesian networks and their corresponding relationship weights [53]. Finally, CompositeView could be applied to large neural network pipelines, which tend to become black boxes with layers upon layers of weights and abstraction [54]. CompositeView, it its current state, may not be able to visualize the results of all possible permutations of the aforementioned analytical techniques; however, due to its inherent flexibility, CompositeView can be adapted to do so with minimal required changes by the user.

#### 4.3.3. Other Applications

As shown in the sample data visualizations in Section 3.2, CompositeView can be adapted to a wide variety of disparate data sets that have a mixture of quantitative and categorical attributes where the user wishes to visualize connections. The choice of domain is flexible, as seen in the sample data analyzed in Section 3.2, and can range from healthcare to business to financial models and more.

#### 4.3.4. Future Work

CompositeView has the potential to improve in three specific areas: optimization, flexibility, and accessibility. Optimization will be dataset-dependent. Thus, further exposure to additional data sets will identify new ways to further optimize speed and efficiency. Additionally, flexibility, in the form of greater software compatibility, is an important next step. Currently, CompositeView is designed to work with the Windows 10 operating system and Google Chrome or Microsoft Edge. Occasional problems involving other operating systems, specifically macOS, along with some browsers, such as Firefox, were noted. Increasing compatibility to additional systems and browsers may entail replacing Dash as the main framework for the application. However, the likely incurred negative trade-off with replacing Dash would be loss of accessibility. Finally, user accessibility can be improved through feature expansion and the introduction of a dedicated package and hosted web application. Additional feature expansion would increase accessibility and customization for non-Python users. For example, new dropdown components would allow users to select different combination methods for viewing and adjusting the composite score without changing the source code. Beyond a hosted web application, packaging CompositeView with more intuitive modules and code integration features will allow it to better integrate itself with existing analytical pipelines, network-based or otherwise.

## 5. Conclusions

In conclusion, with increasingly complex data sets and analysis, there is a need for improved visualization tools. Data, and the algorithms used to analyze them, are only as good as the insight they provide. Information overload impedes human data analysts’ ability to visualize complex results—a problem that motivated the development of CompositeView. CompositeView is an open-source Python-based application that integrates with Cytoscape Dash to enable interactive user manipulation of visualized data and corresponding calculated composite scores. CompositeView was originally built to interpret the results generated by SemNet 2.0, a novel literature-based discovery tool that utilizes network analysis and graph theory to gain insight from text-mined biomedical literature. The addition of CompositeView to the SemNet pipeline was necessary to visualize and meaningfully interpret complex, multi-target simulation results. However, CompositeView was generalized to view composite scores for any network or non-network data set that fits its data input format. The ability to interactively change the complexity of the graph and calculate corresponding composite scores improves the human analyst’s ability to expediently visualize, extract, and interpret actionable insight. CompositeView works under the core tenets of flexibility and accessibility, giving users of the tool the ability to easily and efficiently adapt it to their respective needs for both network and non-network data. The present study showed that CompositeView was able to improve analysis for three disparate case studies: a SemNet 2.0 network simulation for literature-based discovery, a Human Development Index data set, and the Framingham cardiovascular study. Improvements to CompositeView are ongoing, with updates pushed to the corresponding GitHub repository.

## Figures and Tables

**Table 1.** The simplest of the three data examples described in Section 2.1.3. These data are visualized in Figure 2a.

source_node	target_node	edge_value
S4	T1	4
S3	T1	3
S2	T1	2
S1	T1	1

**Table 2.** More complex, but still relatively simple data from the second example described in Section 2.1.3. These data are visualized in Figure 2b.

source_node	target_node	edge_value
S3	T1	3
S3	T2	4
S4	T1	4
S4	T2	2
S1	T1	1
S1	T2	3
S2	T1	2
S2	T2	1

**Table 3.** The full input data format, populated with data from the third example described in Section 2.1.3. These data are visualized in Figure 2c.

source_id	source_name	source_type	target_id	target_name	target_type	edge_value
S3_2	S3	G2	T1_id	T1	T	3
S3_2	S3	G2	T2_id	T2	T	3
S3_2	S3	G2	T3_id	T3	T	4
S3_1	S3	G1	T1_id	T1	T	3
S3_1	S3	G1	T2_id	T2	T	4
S4_1	S4	G1	T1_id	T1	T	4
S4_1	S4	G1	T2_id	T2	T	2
S1_1	S1	G1	T1_id	T1	T	1
S1_1	S1	G1	T2_id	T2	T	3
S2_1	S2	G1	T1_id	T1	T	2
S2_1	S2	G1	T2_id	T2	T	1

**Table 4.** A display of the first input data example manipulated to fit CompositeView’s final data input format.

source_id	source_name	source_type	target_id	target_name	target_type	edge_value
S4_id	S4	G1	T1_id	T1	T	4
S3_id	S3	G1	T1_id	T1	T	3
S2_id	S2	G1	T1_id	T1	T	2
S1_id	S1	G1	T1_id	T1	T	1

## Data Availability

CompositeView is publicly accessible and maintained on GitHub https://github.com/pathology-dynamics/composite_view; accessed on 17 April 2022.
